# Remodeling Intestinal Microbiota Alleviates Severe Combined Hyperlipidemia-Induced Nonalcoholic Steatohepatitis and Atherosclerosis in LDLR^-/-^ Hamsters

**DOI:** 10.34133/research.0363

**Published:** 2024-04-29

**Authors:** Guolin Miao, Jiabao Guo, Wenxi Zhang, Pingping Lai, Yitong Xu, Jingxuan Chen, Lianxin Zhang, Zihao Zhou, Yufei Han, Gonglie Chen, Jinxuan Chen, Yijun Tao, Lemin Zheng, Ling Zhang, Wei Huang, Yuhui Wang, Xunde Xian

**Affiliations:** ^1^ Institute of Cardiovascular Sciences, State Key Laboratory of Vascular Homeostasis and Remodeling, School of Basic Medical Sciences, Peking University, Beijing, China.; ^2^ Beijing Key Laboratory of Cardiovascular Receptors Research, Peking University Third Hospital, Beijing, China.

## Abstract

Combined hyperlipidemia (CHL) manifests as elevated cholesterol and triglycerides, associated with fatty liver and cardiovascular diseases. Emerging evidence underscores the crucial role of the intestinal microbiota in metabolic disorders. However, the potential therapeutic viability of remodeling the intestinal microbiota in CHL remains uncertain. In this study, CHL was induced in low-density lipoprotein receptor-deficient (LDLR^-/-^) hamsters through an 8-week high-fat and high-cholesterol (HFHC) diet or a 4-month high-cholesterol (HC) diet. Placebo or antibiotics were administered through separate or cohousing approaches. Analysis through 16S rDNA sequencing revealed that intermittent antibiotic treatment and the cohousing approach effectively modulated the gut microbiota community without impacting its overall abundance in LDLR^-/-^ hamsters exhibiting severe CHL. Antibiotic treatment mitigated HFHC diet-induced obesity, hyperglycemia, and hyperlipidemia, enhancing thermogenesis and alleviating nonalcoholic steatohepatitis (NASH), concurrently reducing atherosclerotic lesions in LDLR^-/-^ hamsters. Metabolomic analysis revealed a favorable liver lipid metabolism profile. Increased levels of microbiota-derived metabolites, notably butyrate and glycylglycine, also ameliorated NASH and atherosclerosis in HFHC diet-fed LDLR^-/-^ hamsters. Notably, antibiotics, butyrate, and glycylglycine treatment exhibited protective effects in LDLR^-/-^ hamsters on an HC diet, aligning with outcomes observed in the HFHC diet scenario. Our findings highlight the efficacy of remodeling gut microbiota through antibiotic treatment and cohousing in improving obesity, NASH, and atherosclerosis associated with refractory CHL. Increased levels of beneficial microbiota-derived metabolites suggest a potential avenue for microbiome-mediated therapies in addressing CHL-associated diseases.

## Introduction

The increased prevalence of metabolic diseases, encompassing obesity, nonalcoholic fatty liver disease (NAFLD), type 2 diabetes, and cardiovascular diseases (CVDs), imposes a substantial global healthcare burden, profoundly impacting human safety and well-being [1]. Hyperlipidemia, influenced by genetic and environmental factors, has emerged as a pivotal contributor to these metabolic diseases. Of particular note is combined hyperlipidemia (CHL), marked by concurrent hypercholesterolemia and hypertriglyceridemia, standing out as the predominant form of dyslipidemia [2]. The continuous rise in morbidity among patients with metabolic diseases linked to CHL underscores the imperative to enhance the quality of life for these individuals [3]. Despite the widespread use of lipid-lowering therapies, the global prevalence of hyperlipidemia and its associated metabolic diseases persists at alarming levels, necessitating a deeper understanding of mechanisms governing systemic lipid metabolism and homeostasis. The overarching goal is to innovate strategies for effectively treating CHL-associated metabolic diseases.

The intestinal microbiota, in its symbiotic relationship with the host, contributes nutrients and energy through the metabolization of dietary components [4]. Various studies propose the gut microbiome as a substantial environmental factor influencing the progression of multiple diseases, thus presenting a potential drug target [5,6]. Notably, microbiota dysbiosis in obesity has been linked to reduced efficacy of statin treatment, with statins shown to modulate the gut microbiota and reduce body mass index in clinical patients [7]. Similarly, metformin, a hyperglycemia-lowering drug, has been found to alter the gut microbiota community in both humans and mice [8,9], suggesting that the drug’s hyperglycemia-lowering action may result from modulating the gut microbiota population. Moreover, microbiota-related metabolites, such as bile acids, lipopolysaccharide (LPS), and short-chain fatty acids (SCFAs), have been reported to play a role in regulating hyperlipidemia [10]. For instance, SCFAs can induce the release of hormones by binding to G-protein-coupled receptors, leading to increased satiety and reduced food intake [11]. Additionally, alterations in gut microbiota profiles have been observed in CHL patients [10], emphasizing the importance of further investigations to characterize the contributions of gut microbiota and microbiota-related metabolites to CHL and its associated abnormalities.

It is well known that the metabolic process differs between humans and mice, especially lipid metabolism [12]. Wild-type (WT) mice are resistant to diet-induced hyperlipidemia, while mice lacking low-density lipoprotein receptor (LDLR) only exhibit hypercholesterolemia, failing to replicate the clinical characteristics of CHL patients [13]. Interestingly, previous research demonstrated that high-fat diet (HFD)-fed WT golden Syrian hamsters display hyperlipidemia, insulin resistance, and hepatic steatosis, suggesting the hamster model’s predisposition to HFD-induced metabolic disorders akin to those observed in humans [14]. Importantly, homozygous LDLR-deficient (LDLR^-/-^) hamsters exhibit both hypercholesterolemia and hypertriglyceridemia, making them a more appropriate animal model for studying CHL-related metabolic diseases.

In this study, we explored the impact of modulating gut microbiota through intermittent antibiotic treatment and subsequent transfer via cohousing. Additionally, we investigated the potential benefits of supplementation with beneficial microbial metabolites. Our objective was to improve the abnormal metabolic phenotypes associated with severe CHL in both high-fat and high-cholesterol (HFHC) and high-cholesterol (HC) diet-induced LDLR^-/-^ hamsters.

## Results

### Modulation of gut microbiota mitigates obesity and metabolic abnormalities induced by HFHC diet

**Fig. 1.**
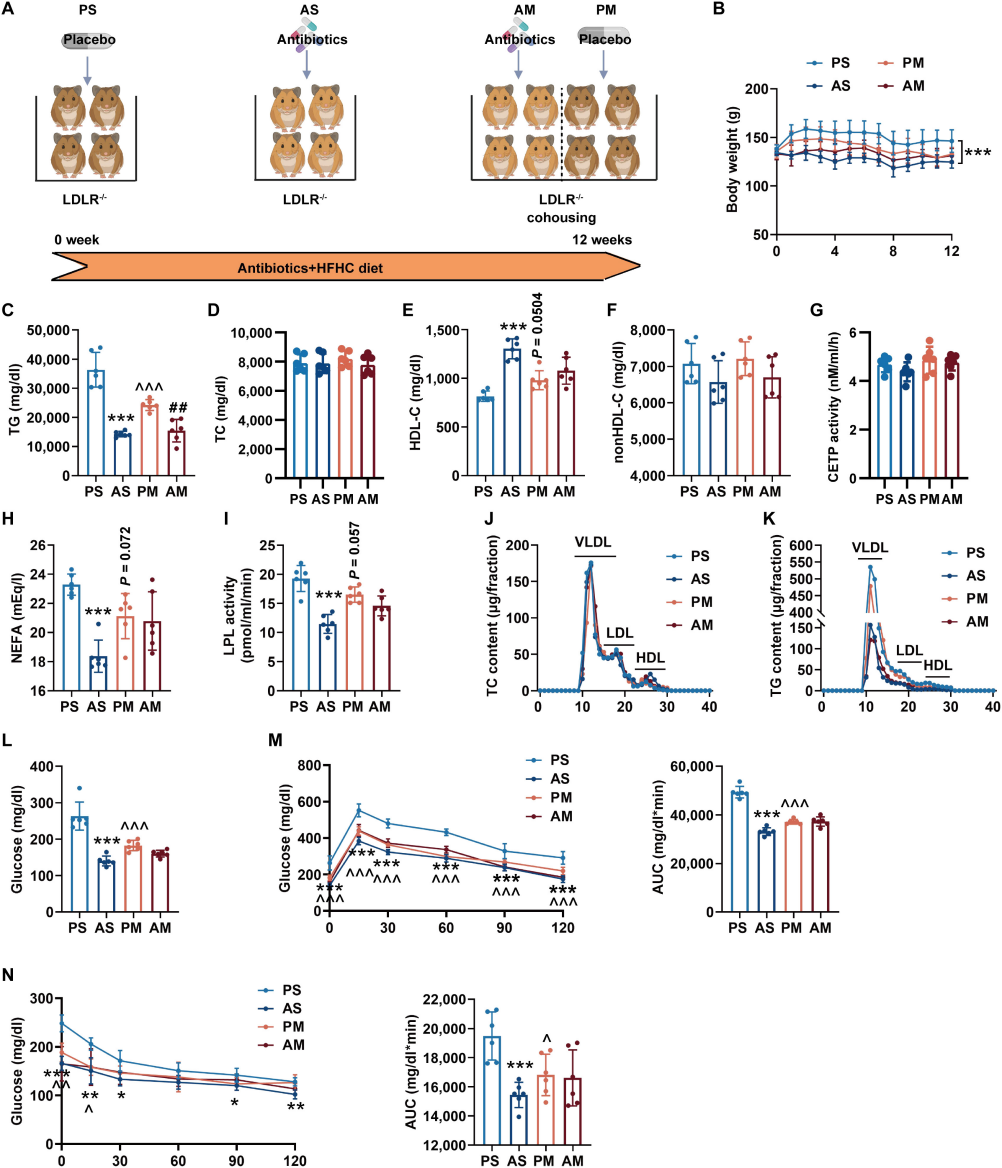
Antibiotics treatment generated a favorable metabolic profile in LDLR^-/-^ hamsters. (A) Schematic diagram of the experiment. The LDLR^-/-^ hamsters fed HFHC diet were given a placebo or antibiotics for 8 weeks by a separate or cohousing approach (*n* = 6/group). PS, LDLR^-/-^ hamsters given placebo were separately housed; AS, LDLR^-/-^ hamsters with antibiotic treatment were separately housed; PM, LDLR^-/-^ hamsters given placebo were cohoused with LDLR^-/-^ hamsters given antibiotics; AM, LDLR^-/-^ hamsters given antibiotics were cohoused with LDLR^-/-^ hamsters given placebo. (B) The body weight of LDLR^-/-^ hamsters. (C to H) Determination of plasma TG (C), cholesterol (TC) (D), HDL-C (E), nonHDL-C (F), CETP activity (G), and nonesterified free fatty acids (NEFA) (H) in LDLR^-/-^ hamsters after overnight fasting (*n* = 6/group). (I) Plasma LPL activity. (J and K) The distribution of cholesterol (J) and TG (K) in pooled plasma samples from the indicated groups after 8-week HCHF diet feeding (*n* = 6/group). (L) Determination of plasma glucose. (M and N) In the fourth week, glucose tolerance tests (M) and insulin tolerance tests (N) were performed. Data are expressed as means ± SEM, analyzed by 2-way ANOVA using Prism 8.0. *P < 0.05, **P < 0.01, ***P < 0.001 AS vs PS; ^^^*P* < 0.05, ^^^^*P* < 0.01, ^^^^^*P* < 0.001 PM vs PS; ^##^*P* < 0.01 AM vs PM. VLDL, very-low-density lipoprotein.

Recently, various clinical and experimental studies have underscored the pivotal role of gut microbiota in the development of metabolic syndrome. To investigate the potential benefits of modulating gut microbiota in severe CHL disease, male LDLR^-/-^ hamsters on an HFHC diet were intermittently treated with antibiotics or placebo, utilizing both separate and cohousing approaches (Fig. 1A). Firstly, we performed 16S rDNA gene sequencing on stools from the indicated 4 groups of LDLR^-/-^ hamsters (PS, LDLR^-/-^ hamsters given placebo were separately housed; AS, LDLR^-/-^ hamsters with antibiotic treatment were separately housed; PM, LDLR^-/-^ hamsters given placebo were cohoused with LDLR^-/-^ hamsters given antibiotics; AM, LDLR^-/-^ hamsters given antibiotics were cohoused with LDLR^-/-^ hamsters given placebo). The Simpson indexes indicated no substantial differences in bacterial richness among the groups (*P* > 0.05) (Fig. S1A). However, weighted Unifrac principal component analysis (PCA) revealed compositional discrimination in gut microbiota between placebo- and antibiotics-treated LDLR^-/-^ hamsters using both separate and cohousing approaches (Fig. S1B). Near discriminant analysis identified distinct dominant bacteria genera, with butyrate (BA)-producing bacteria *Blautia* being most abundant in LDLR^-/-^ hamsters from the AS group (Fig. S1C).

**Table.** Nonstandard abbreviations and acronyms

ALT	Alanine aminotransferase
AST	Aspartate transaminase
BA	Butyrate
BAT	Brown adipose tissue
CHL	Combined hyperlipidemia
CVD	Cardiovascular disease
FPLC	Fast protein liquid chromatography
GG	Glycylglycine
LDLR	Low-density lipoprotein receptor
LPL	Lipoprotein lipase
NEFA	Nonesterified fatty acid
NAFLD	Nonalcoholic fatty liver disease
NASH	Nonalcoholic steatohepatitis
PCA	Principal component analysis
TC	Total cholesterol
TG	Triglyceride
UCP1	Uncoupling protein 1
WAT	White adipose tissue

Subsequent observations showed a reduced body weight gain in the AS group compared to the PS group after 8 weeks of HFHC-diet feeding (Fig. 1B). Additionally, hamsters receiving antibiotics exhibited higher body temperature and reduced fat weight/body weight ratios in subcutaneous and epididymal white adipose tissue (sWAT and eWAT) (Fig. S2A and B). Histological analyses revealed eWAT and brown adipose tissue (BAT) of LDLR^-/-^ hamsters in the AS group were small and multilocular lipid droplets (Fig. S2C, D, and F). Immunohistochemical analysis showed that macrophage infiltration was decreased in eWAT, while uncoupling protein 1 (UCP1) expression was increased in BAT of LDLR^-/-^ hamsters from the AS group (Fig. S2E and G). Thus, the antibiotic treatment caused pronounced morphological and functional alterations in adipocytes.

Although total cholesterol (TC) and non-high-density lipoprotein cholesterol (nonHDL-C) levels showed no substantial differences between AS and PS groups (Fig. 1D and F), the plasma triglyceride (TG), HDL-C, glucose, and nonesterified fatty acid (NEFA) levels were substantially descended, accompanied with hypoactivity of lipoprotein lipase (LPL) (Fig. 1C, E, H, I, and L) in AS group when compared to the PS group. Given the pivotal role of cholesteryl ester transfer protein (CETP) in both HDL-C and TG metabolism, we measure CETP activity; no substantial differences were observed among the 4 experimental groups (Fig. 1G). Lipid distribution analyzed by fast protein liquid chromatography (FPLC) demonstrated that cholesterol content was increased in the HDL fractions in LDLR^-/-^ hamsters of the AS group compared with LDLR^-/-^ hamsters of the PS group, with no apparent changes in very-low-density lipoprotein and low-density lipoprotein fractions (Fig. 1J). Moreover, the TG concentration in the very-low-density lipoprotein fractions was visibly reduced in the AS-group animals (Fig. 1K). Glucose tolerance test and insulin tolerance test results demonstrated substantial improvements in glucose intolerance and insulin resistance after antibiotic treatment (Fig. 1M and N). Furthermore, fecal microbiota transplantation from antibiotics-treated (AM) hamsters to placebo-treated (PM) hamsters via cohousing protected against HFHC diet-induced metabolic abnormalities, emphasizing the beneficial effects of gut microbiota modulation.

### Modulation of gut microbiota alleviated nonalcoholic steatohepatitis and atherosclerosis

**Fig. 2.**
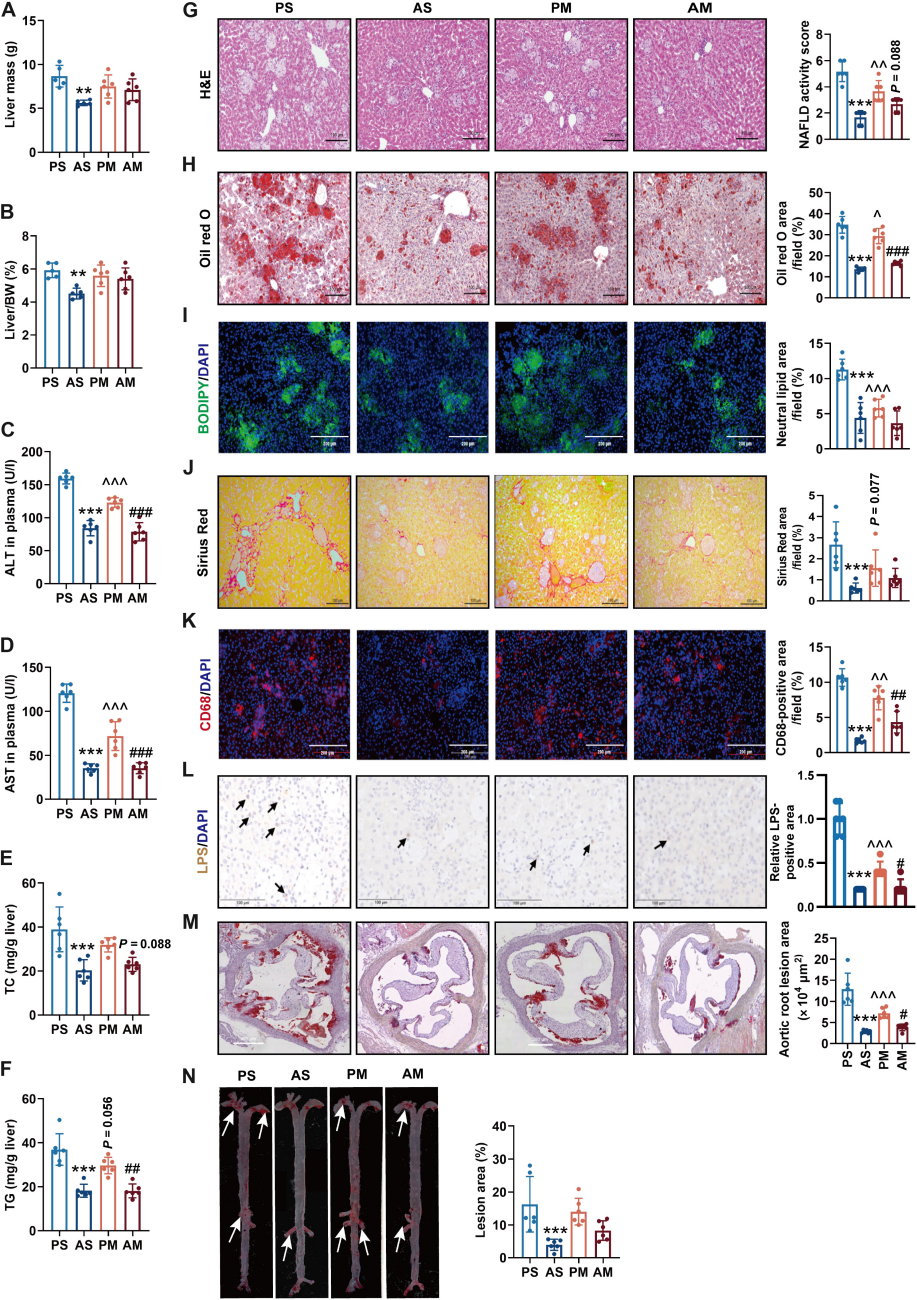
Antibiotics treatment protected from HFHC diet-induced hepatic injury and atherosclerosis in LDLR^-/-^ hamsters. (A) The liver weight. (B) The ratio of liver weight to body weight (BW). (C) Plasma ALT levels. (D) Plasma AST levels. (E) The content of TC in the liver. (F) The content of total TG in the liver. (G) Hematoxylin and eosin (H&E) staining analyses of liver sections to reveal the histopathology changes. (H) Oil red O staining to specially indicate lipid droplets. (I) BODIPY staining to specially label neutral lipids. The nucleus was stained by DAPI. (J) The disposed of collagen fibers were stained by Sirius Red. (K) Frozen liver sections were stained by CD68 antibody to display the aggregated Kupffer cells. The nucleus was stained by DAPI. (L) Immunohistochemical staining of LPS in liver to reveal the translocation of microbiota from the gut. Positive immunoreactivity was observed as a brown precipitate. (M) Representative oil red O staining of the aortic root. (N) Representative oil red O staining of the whole aorta. White arrows indicate positive staining. Data are expressed as means ± SEM, analyzed by 2-way ANOVA using Prism 8.0. **P < 0.01 and ***P < 0.001 AS vs PS; ^^^*P* < 0.05, ^^^^*P* < 0.01, ^^^^^*P* < 0.001 PM vs PS; ^#^*P* < 0.05, ^##^*P* < 0.01, ^###^*P* < 0.001 AM vs PM. PS, LDLR^-/-^ hamsters given placebo were separately housed; AS, LDLR^-/-^ hamsters with antibiotic treatment were separately housed; PM, LDLR^-/-^ hamsters given placebo were cohoused with LDLR^-/-^ hamsters given antibiotics; AM, LDLR^-/-^ hamsters given antibiotics were cohoused with LDLR^-/-^ hamsters given placebo.

Obesity and abnormal lipid metabolism are major risk factors for NAFLD. Our investigation then focused on assessing liver function parameters in LDLR^-/-^ hamsters subjected to an HFHC diet. We observed that the liver weight and the liver weight to body weight ratio were reduced in AS-group hamsters compared with hamsters of the PS group (Fig. 2A and B). Substantially decreased plasma alanine aminotransferase (ALT) and aspartate transaminase (AST) levels, as well as liver TC and TG levels, indicated substantial improvement in liver injury induced by the HFHC diet in LDLR^-/-^ hamsters (Fig. 2C to F).

Pathological histology analysis demonstrated a marked alleviation of nonalcoholic steatohepatitis (NASH) phenotypes (steatosis, inflammation, and ballooning) in the AS group, accompanied by a reduced NAFLD activity score (Fig. 2G). Moreover, lipid accumulation, as indicated by oil red O and BODIPY dye staining, was notably reduced in hamsters receiving antibiotic treatment (Fig. 2H and I). Sirius Red staining for fibrosis revealed a substantial reduction in collagen deposition surrounding macrovesicular lipid droplets in the AS group (Fig. 2J). Antibiotics also interrupted Kupffer cell aggregation and LPS accumulation (Fig. 2K and L). Consistent with the pathological observations, transcriptional analysis indicated that the genes involved in cholesterol efflux were up-regulated (Fig. S3A). Notably, there was a substantial down-regulation in hepatic expression of genes related to cholesterol synthesis, secretion, inflammation, and fibrosis (Fig. S3B and D). Meanwhile, the expression levels of genes governing fatty acid uptake, synthesis, and oxidation were moderately reduced (Fig. S3C), which could be due to the suppression of adipocyte hypertrophy by antibiotic treatment. Moreover, gut microbiota transfer via cohousing replicated protective effects on NASH in LDLR^-/-^ hamsters of the PM group compared with the PS group (Fig. 2A to L and Fig. S3A to D). Circulating immune cell analysis revealed no substantial differences in the number and percentage of monocytes, granulocytes, and lymphocytes in the host blood following intermittent antibiotic treatment and the cohousing approach (Fig. S4A to G).

**Fig. 3.**
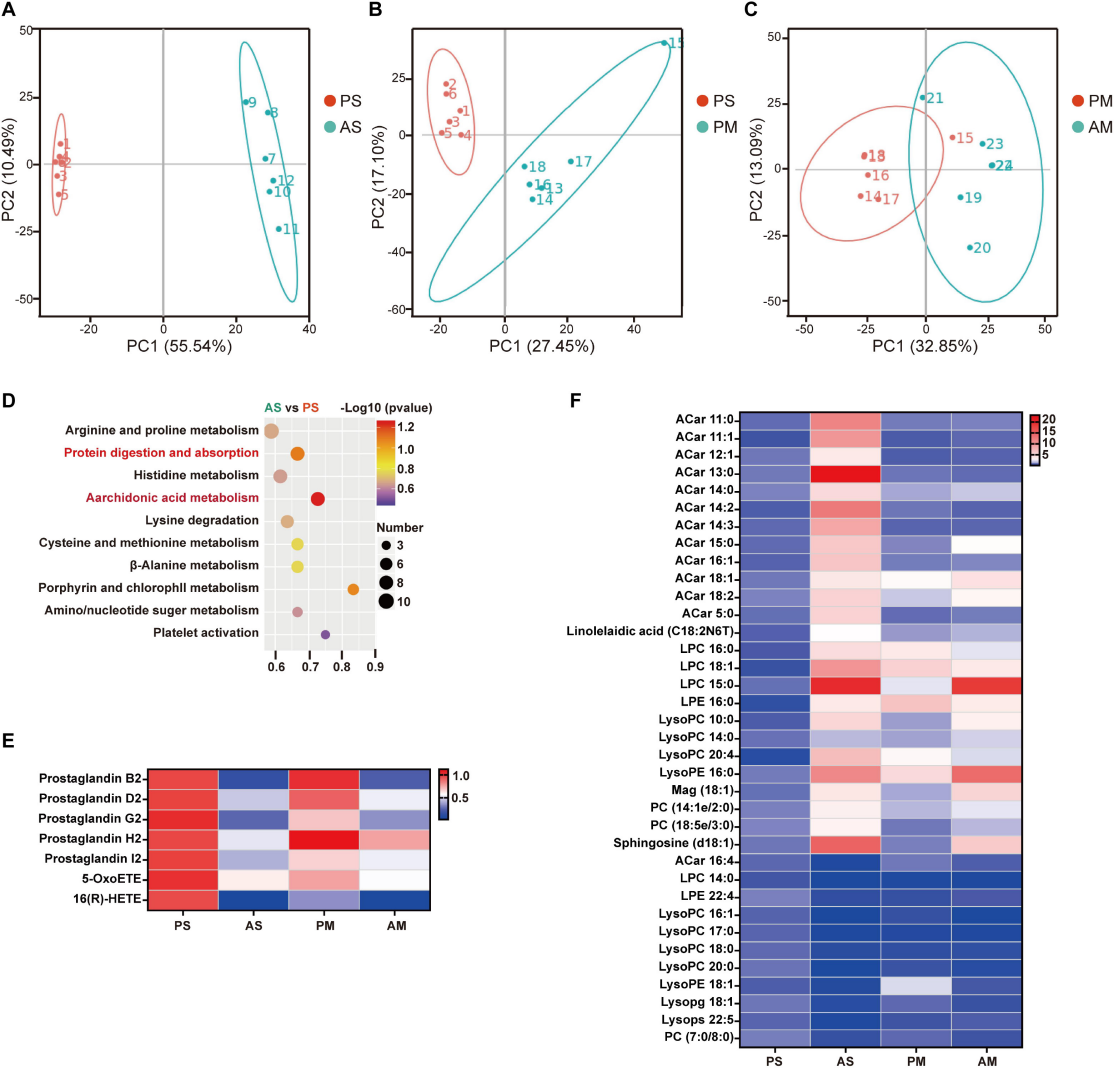
Antibiotics treatment improved the metabolism profile of the liver in LDLR^-/-^ hamsters. The LDLR^-/-^ hamsters fed HFHC diet were given a placebo or antibiotics for 8 weeks by a separate or cohousing approach (*n* = 6/group). A nontargeted metabolomics approach was employed to elucidate these mechanisms by identifying metabolic changes associated with NASH in LDLR^-/-^ hamsters. (A to C) PCA of liver metabolites between AS and PS groups, PM and PS groups, and AM and PM groups LDLR^-/-^ hamsters. (D) Kyoto Encyclopedia of Genes and Genomes enrichment pathway of nontargeted metabolomics data between AS and PS groups LDLR^-/-^ hamsters' liver. (E and F) Heat map generated from nontargeted metabolomics data of LDLR^-/-^ hamster's liver involved in arachidonic acid (E) and other lipids (F). Blue and red colors indicate down-regulation and up-regulation, respectively. PS, LDLR^-/-^ hamsters given placebo were separately housed; AS, LDLR^-/-^ hamsters with antibiotic treatment were separately housed; PM, LDLR^-/-^ hamsters given placebo were cohoused with LDLR^-/-^ hamsters given antibiotics; AM, LDLR^-/-^ hamsters given antibiotics were cohoused with LDLR^-/-^ hamsters given placebo.

To study how gut microbiota restruction altered hepatic injury, we performed metabolic profiling of the livers of 4 groups of LDLR^-/-^ hamsters. Metabolic profiling of liver tissues through PCA highlighted substantial differences attributable to antibiotic treatment and cohousing (Fig. 3A to C). Arachidonic acid metabolism was a critical pathway altered in LDLR^-/-^ hamsters for the AS group (Fig. 3D). Prostaglandin B2, D2, G2 H2 I2, 5-OxoETE, and 16(R)-HETE were down-regulated metabolites in LDLR^-/-^ hamsters of the AS group (Fig. 3E). Also, antibiotics treatment exhibited increased levels of beneficial lipid metabolism markers, including acylcarnitines (Acar), linolelaidic acid, lysophosphatidylcholine, lysophosphatidylethanolamines, phosphatidylcholine, and sphingosine in liver accompanied with decreased glycerophospholipids (lysopc, lysope, lysopg, and lysops) (Fig. 3F).

Of note, LDLR^-/-^ hamster was an ideal small rodent animal model used for studying human atherosclerosis, and metabolic disorders are highly linked to atherogenesis. Thus, we investigated the effects of antibiotic treatment or gut microbiota transfer on atherosclerotic development. Our oil red O staining data disclosed that the atherosclerotic lesion areas in both the aortic roots (Fig. 2M) and whole aortas (Fig. 2N) were markedly reduced in antibiotic treatment and cohousing LDLR^-/-^ hamsters.

To better explore the protective effects of reshaping intestinal microbiome by antibiotics on hyperlipidemia-associated atherosclerosis and NAFLD in CHL patients, we subjected LDLR^-/-^ hamsters to an HC diet (Fig. S5A). In line with the findings from the severe combined hyperlipidemic LDLR^-/-^ hamster model mentioned earlier, antibiotic treatment not only substantially curtailed body weight gain (Fig. S5B) but also led to a reduction in plasma TC, TG, and nonHDL-C levels (Fig. S5C, D, and F), while elevating HDL-C levels (Fig. S5E), thus then resulting in a decrease in atherosclerotic plaque formation both in the aorta (Fig. S5G and H) and the aortic root (Fig. S5I and J). Furthermore, antibiotic treatment effectively reduced plasma ALT (Fig. S5K) and AST (Fig. S5L) levels, decreased the liver weight/body weight ratio (Fig. S5M), and alleviated hepatic lipid accumulation (Fig. S5N and O), eventually preventing liver injury.

### Antibiotic treatment inhibited intestinal lipid uptake and maintained junction integrity

**Fig. 4.**
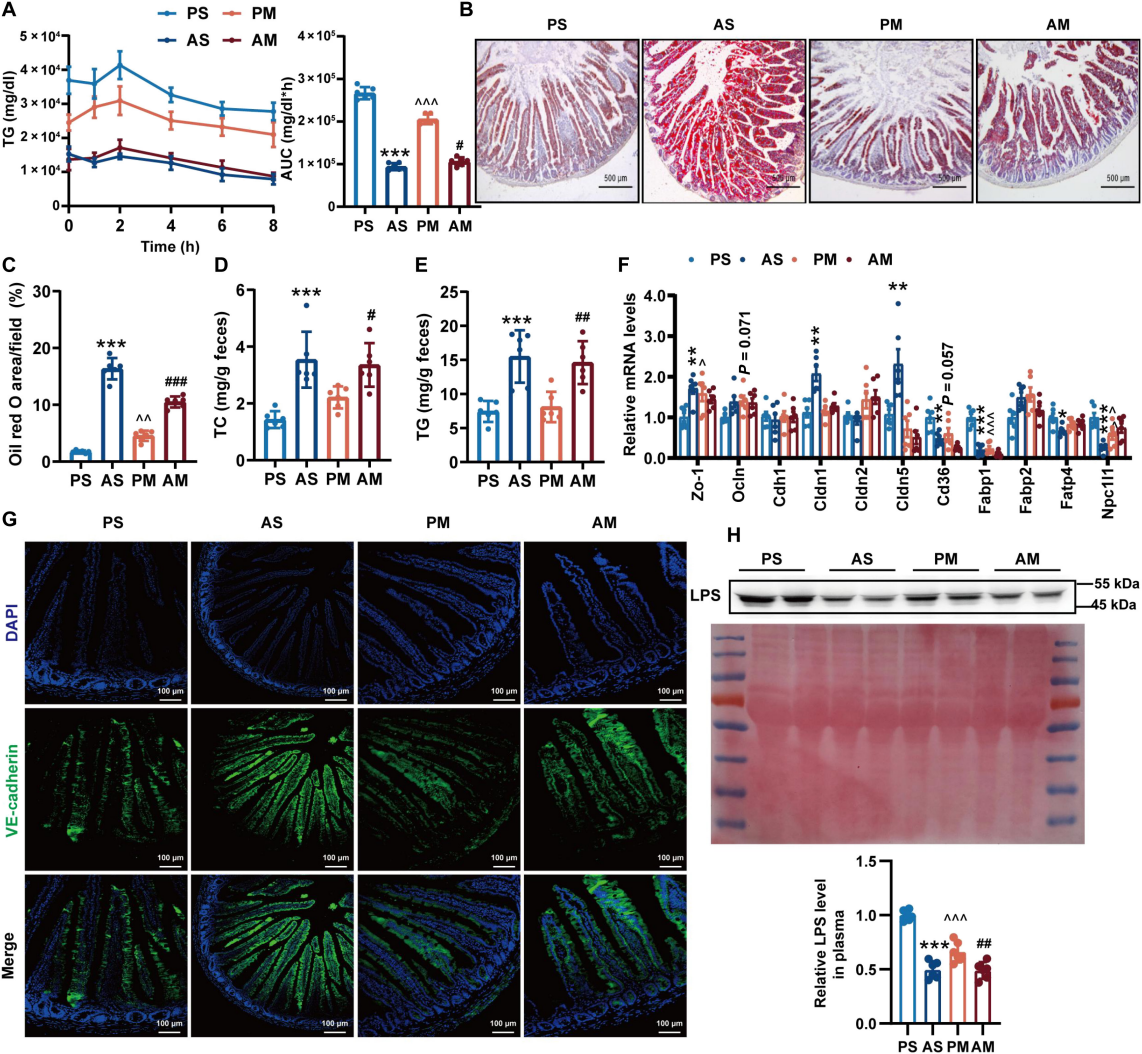
Antibiotic treatment reduced intestinal lipid absorption in HFHC diet-fed LDLR^-/-^ hamsters. (A) Oral lipid tolerance test showing the absorption of TG from the intestine of LDLR^-/-^ hamster by oral gavage of olive oil. *n* = 6/group. (B) Oil red O staining of ileum tissues in LDLR^-/-^ hamsters 6 h after oral gavage of olive oil (10 ml/kg body weight). (C) Quantitative analysis of lipid accumulation in ileum tissue. *n* = 6/group. (D) Feces TC levels. (E) Feces TG levels. (F) mRNA levels of genes associated with mucosal permeability, lipid uptake, and transport in the ileum tissues of LDLR^-/-^ hamsters. (G) Immunofluorescence staining of the zippering junction with VE-cadherin. (H) Plasma LPS content. Data are expressed as means ± SEM, analyzed by 2-way ANOVA using Prism 8.0. *P < 0.05, **P < 0.01 and ***P < 0.001 AS vs PS; ^^^*P* < 0.05, ^^^^*P* < 0.01, ^^^^^*P* < 0.001 PM vs PS; ^#^*P* < 0.05, ^##^*P* < 0.01, ^###^*P* < 0.001 AM vs PM. PS, LDLR^-/-^ hamsters given placebo were separately housed; AS, LDLR^-/-^ hamsters with antibiotic treatment were separately housed; PM, LDLR^-/-^ hamsters given placebo were cohoused with LDLR^-/-^ hamsters given antibiotics; AM, LDLR^-/-^ hamsters given antibiotics were cohoused with LDLR^-/-^ hamsters given placebo. AUC, area under the curve.

The gastrointestinal system plays a pivotal role in nutrient digestion and absorption. Our investigation revealed a substantial reduction in postprandial plasma TG levels, while lipids were prominently retained in the ileum tissue of LDLR^-/-^ hamsters in the AS and PM groups compared to the PS group, respectively (Fig. 4A to C). Consistent with this, there was a notable increase in the contents of TC and TG in feces from the AS and PM groups compared to the PS group (Fig. 4D and E).

The intestinal epithelial barrier serves as the initial defense against luminal environmental factors. A loss of barrier integrity allows the translocation of luminal antigens (microbes and toxins) via the mucosa to access the whole body and subsequently destroys the gut mucosal homeostasis, coinciding with increased susceptibility to systemic inflammation. In response to antibiotic treatment, mRNA levels of tight junction markers ZO-1 and Ocln and Cdh1/5 were visibly up-regulated in LDLR^-/-^ hamsters from both AS and PM groups (Fig. 4F). Notably, the expression levels of genes involved in lipid absorption (CD36 and NPC1L1) and transport (FABP1 and FATP4) in ileum tissue were substantially decreased post-antibiotic treatment (Fig. 4F). Immunofluorescence staining of zippering junctions with vascular endothelial (VE)-cadherin supported these findings, revealing more continuous junctions in the antibiotic-treated groups (Fig. 4G). This structural enhancement indicates a strengthened barrier function, effectively inhibiting chylomicron uptake. The reduction in circulating LPS levels in LDLR^-/-^ hamsters following intermittent antibiotic treatment and cohousing further underscores improved gut integrity (Fig. 4H). Together, these analyses highlight the positive impact of combined interventions on reinforcing intestinal integrity and impeding paracellular permeability, demonstrating that both antibiotic treatment and cohousing maintain a healthy gut environment in LDLR^-/-^ hamsters.

### Reshaping gut microbiota altered microbiota metabolites

**Fig. 5.**
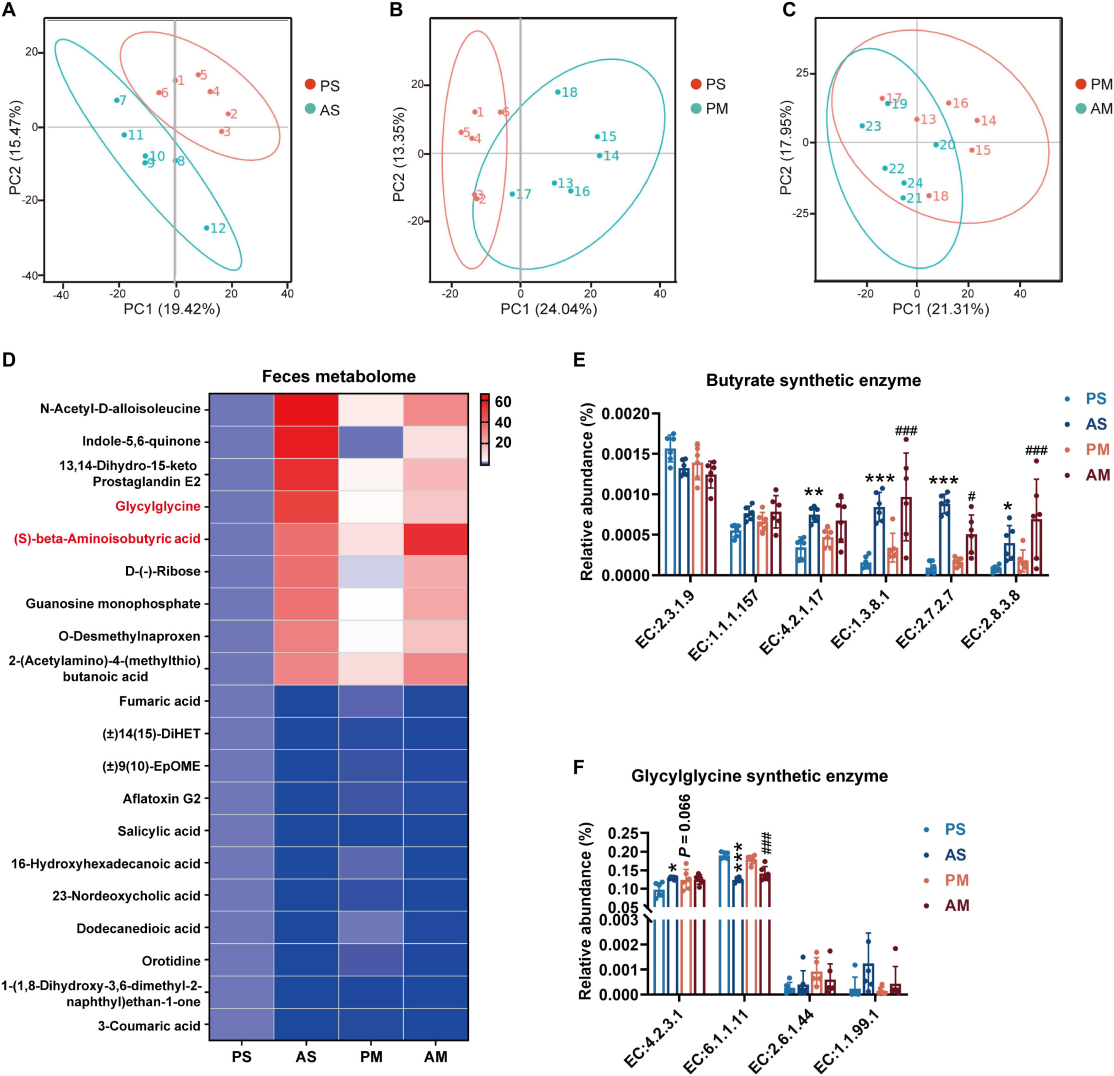
Effects of antibiotic treatment on fecal microbiota metabolites in HFHC diet-fed LDLR^-/-^ hamsters. The LDLR^-/-^ hamsters fed HFHC diet were given a placebo or antibiotics for 8 weeks by a separate or cohousing approach (*n* = 6/group). A nontargeted metabolomics approach was performed to detect fecal microbial metabolites in LDLR^-/-^ hamsters. (A to C) PCA of microbiota metabolites between AS and PS groups (A), PM and PS groups (B), and AM and PM groups (C) LDLR^-/-^ hamsters. (D) Heat maps of substantially different fecal microbial metabolites in LDLR^-/-^ hamster. (E and F) BA (E) and GG (F) synthesis-related enzymes abundance in fecal samples. Data are expressed as means ± SEM, analyzed by 2-way ANOVA using Prism 8.0. *P < 0.05, **P < 0.01 and ***P < 0.001 AS vs PS ^#^*P* < 0.05, ^#^*P* < 0.05, ^###^*P* < 0.001 AM vs PM. PS, LDLR^-/-^ hamsters given placebo were separately housed; AS, LDLR^-/-^ hamsters with antibiotic treatment were separately housed; PM, LDLR^-/-^ hamsters given placebo were cohoused with LDLR^-/-^ hamsters given antibiotics; AM, LDLR^-/-^ hamsters given antibiotics were cohoused with LDLR^-/-^ hamsters given placebo.

The pathogenic role of gut microbiota in the development of hyperlipidemia has been uncovered through fecal microbiota transplantation experiments conducted on germ-free mice [10]. Furthermore, the effector mechanism of microbiota-related metabolites, including bile acids, LPS, and SCFAs, in regulating hyperlipidemia has been partially elucidated [15,16]. Gas chromatography-mass spectrometry analysis of cecal samples revealed that, in line with the observed changes in intestinal bacterial composition, antibiotic treatment and cohousing substantially reconstructed microbiota-related metabolites in LDLR^-/-^ hamsters (Fig. 5A to C). Specifically, the concentrations of β-aminoisobutyric acid and glycylglycine (GG) in the AS and PM groups were elevated (Fig. 5D). Moreover, there was an increase in the levels of BA synthesis- and GG synthesis-related enzymes (Fig. 5E and F). According to the above, the cohousing approach could transmit antibiotic treatment’s anti-NASH and anti-atherosclerosis effects. Microbial metabolites BA and GG may be responsible for the indirect benefits of antibiotic treatment and the cohousing approach.

### BA and GG relieved HFHC diet-induced NASH and atherosclerosis

**Fig. 6.**
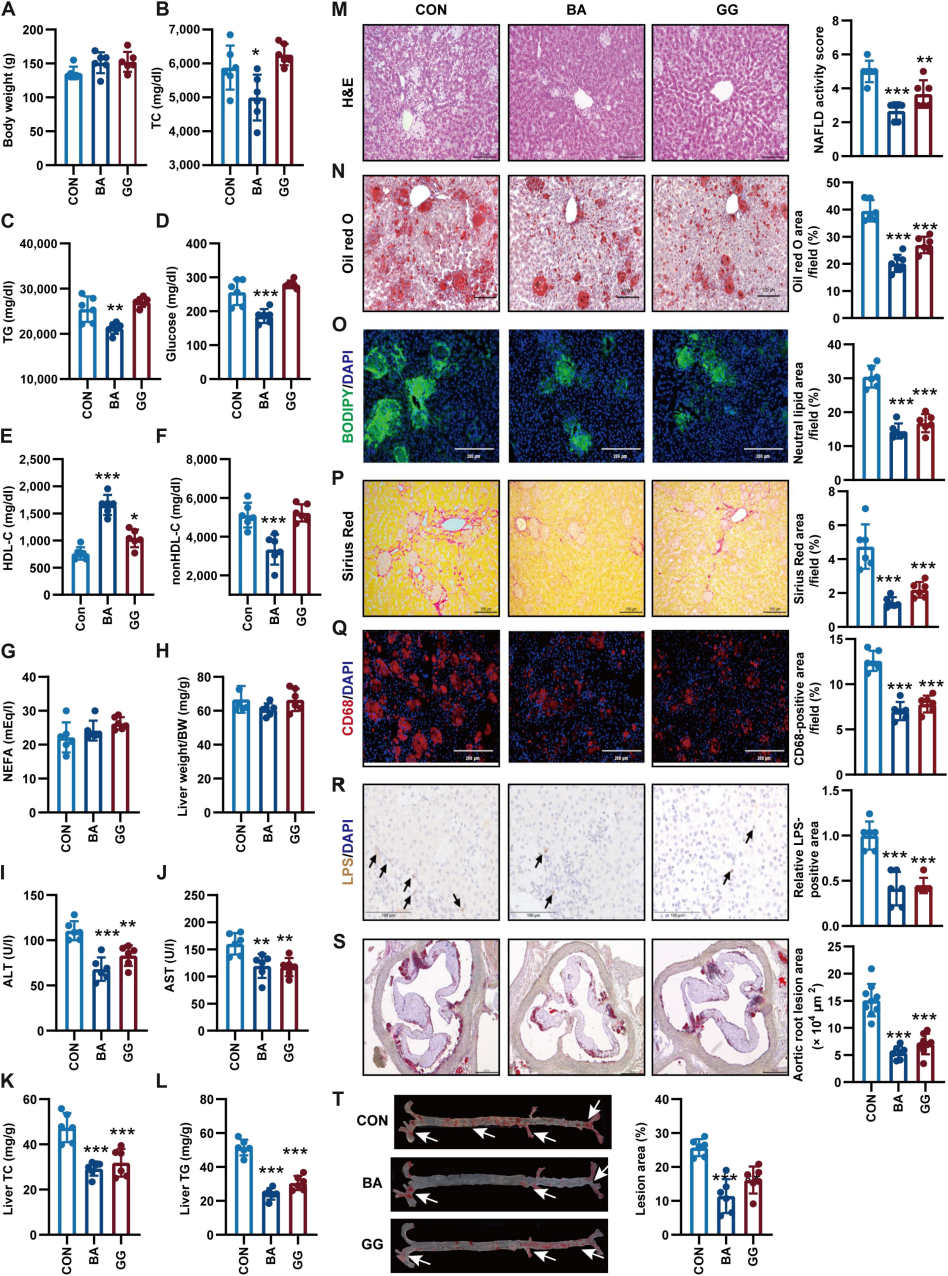
BA and GG treatment alleviated HFHC diet-induced NASH and atherosclerosis in LDLR^-/-^ hamsters. The LDLR^-/-^ hamsters fed an HFHC diet were given sodium BA or GG for 8 weeks (*n* = 6/group). (A) Body weight. (B to G) Determination of plasma cholesterol (TC) (B), TG (C), glucose (D), HDL-C (E), nonHDL-C (F), and nonesterified free fatty acid (NEFA) (G) in LDLR^-/-^ hamsters after overnight fasting (*n* = 6/group). (H) The ratio of liver weight to body weight. (I and J) Plasma ALT (I) and AST (J) levels. (K) The level of TC in the liver. (L) The level of TG in the liver. (M) Hematoxylin and eosin staining analyses of liver sections to reveal the histopathology changes. (N) Oil red O staining of liver sections to specially indicate lipid droplets. (O) BODIPY staining of liver sections to specially label neutral lipids. The nucleus was stained by 4′,6-diamidino-2-phenylindole (DAPI). (P) The disposed collagen fibers were stained by Sirius Red. (Q) Frozen liver sections were stained by CD68 antibody to display the aggregated Kupffer cells. The nucleus was stained by DAPI. (R) Immunohistochemical staining of LPS in liver to reveal the translocation of microbiota from the gut. Positive immunoreactivity was observed as a brown precipitate. (S) Representative oil red O staining of the aortic root. (T) Representative oil red O staining of the whole aorta. White arrows indicate positive staining. Data are expressed as means ± SEM, analyzed by 1-way ANOVA using Prism 8.0. *P < 0.05, **P < 0.01 and ***P < 0.001 BA or GG vs CON. CON, LDLR^-/-^ hamsters were given control water; BA, LDLR^-/-^ hamsters were given sodium butyrate; GG, LDLR^-/-^ hamsters were given glycylglycine.

In evaluating the impact of BA and GG on NASH and atherosclerosis, LDLR^-/-^ hamsters fed an HFHC diet were treated with BA in drinking water, GG through oral administration, and control water (CON) over an 8-week period. While the body weight of LDLR^-/-^ hamsters in the BA and GG groups showed a slight increase, with no substantial difference observed (Fig. 6A), food intake in both groups did not substantially differ from the control group (Fig. S6A). Notably, water intake in the BA group substantially increased compared to the control group, while no substantial change was observed in the GG group (Fig. S6B). Compared with control group, BA reduced plasma TC, TG, glucose, and nonHDL-C levels and increased HDL-C levels, while GG had no effect on these parameters (Fig. 6B to F). NEFA concentration in plasma (Fig. 6G) and the ratio of liver weight to body weight (Fig. 6H) remained unaffected by BA and GG. However, both treatments resulted in reduced levels of plasma ALT (Fig. 6I) and AST (Fig. 6J), as well as the concentration of liver TC (Fig. 6K) and TG (Fig. 6L), indicating substantial improvement in HFHC diet-induced liver injury in LDLR^-/-^ hamsters. Furthermore, BA and GG effectively attenuated hepatocyte ballooning, macrovesicular lipid droplets, fibrosis, macrophage aggregations, and LPS translocation compared to control water (Fig. 6M to R, and Fig. S6C). Additionally, the mRNA levels of genes associated with lipogenesis, inflammation, and fibrosis were down-regulated by both BA and GG (Fig. S6D to F). Furthermore, the atherosclerotic plaques were also markedly reduced (Fig. 6S and T). These results demonstrate that BA and GG have therapeutic effects on alleviating NASH and atherosclerosis in LDLR^-/-^ hamsters with severe CHL.

Similarly, LDLR^-/-^ hamsters were predisposed to an HC diet following BA and GG treatment to assess the protective effects on atherosclerosis and NAFLD (Fig. S7A). BA and GG treatment had no impact on body weight gain (Fig. S7B), HDL-C levels (Fig. S7E), and the liver weight/body weight ratio (Fig. S7I), but it substantially reduced plasma levels of TC, TG, nonHDL-C, ALT, and AST (Fig. S7C, D, and F to H), leading to a notable decrease in atherosclerotic plaque formation both in the aorta (Fig. S7J and K) and at the aortic root (Fig. S7L and M), accompanied by improved hepatic lipid accumulation (Fig. S7N and O).

## Discussion

Extensive evidence suggests that CHL leads to an increased risk of metabolic diseases such as type 2 diabetes, NAFLD, and CVD with higher incidence in men than women, which is attributed to the imbalance between digestion and absorption of nutrients and energy expenditure [17]. Dysfunctional microbiota is highly associated with the perturbation of glucose and lipid metabolism in the host, indicating that the gut microbiota could be a potential therapeutic target for metabolic diseases [18]; however, the precise function and the beneficial role of the gut microbiota in CHL patients with refractory hypercholesterolemia and hypertriglyceridemia have not been fully elucidated, thus limiting the therapeutic applicability to patients with CHL disease who exhibit abnormal microbiota profiles. In the present work, we provide direct evidence that remodeling gut microbiota composition by intermittent antibiotic treatment can generate a favorable lipid metabolism profile in HFHC diet-fed male LDLR^-/-^ hamsters, showing a reduction in TG levels and an increase in HDL-C levels in circulation, accompanied by the reduced inflammatory response. Untargeted metabolomic technique has identified that BA and GG are 2 major metabolites after antibiotic administration. The oral supplementation of BA or GG is sufficient to alleviate the pathological consequence caused by severe CHL largely through inhibiting the inflammation. Our observations imply a link between microbiota composition, lipid metabolism, inflammation, NASH, and atherosclerosis in CHL disease.

Previously independent studies revealed that antibiotic cocktail improved hepatic steatosis in HFD-fed WT mice and hamsters by affecting lipid metabolism [19,20]; however, whether such antibiotic application executes beneficial function in metabolic disease in the context of hyperlipidemia, including CHL disease, has not been fully investigated yet. Herein, we found that intermittent antibiotic treatment could effectively lower circulating TG concentration and increase HDL-C levels in HFHC diet-fed LDLR^-/-^ hamsters. Notably, this lipid lowering effect was independent on the LPL-mediated lipolysis of TRLs because plasma LPL activity was substantially reduced in LDLR^-/-^ hamsters receiving antibiotics. Our observation of TG reduction might be explained by the blockade of intestinal absorption capacity and the increased retention time of dietary lipids in the epithelium of the intestine. Consequentially, the clearance rate of circulating TRLs by LPL was reduced though a feedback loop mechanism due to a decrease in chylomicron-derived TGs secreted by the intestine. Interestingly, unlike TG and HDL, antibiotic treatment had no influence on the plasma levels of TC and nonHDL-C. We speculated that this beneficial effect on lipid metabolism was masked by extreme hyperlipidemia. When LDLR^-/-^ hamsters were challenged with an HC diet containing only 0.05% cholesterol to maintain plasma TC level at ~2000 mg/dl and TG level at ~1500 mg/dl, a marked reduction in TC and nonHDL-C levels was observed with consistently decreased TG and increased HDL levels, suggesting that antibiotic treatment generated a more favorable lipid profile under moderate CHL condition than that observed in LDLR^-/-^ hamsters with severe CHL.

In line with the concept that hypertriglyceridemia has been recognized as an independent risk factor of NAFLD and CVD [21], we found that under the condition of antibiotic treatment, the deposition of TG and TC in the liver was substantially reduced with lower NAFLD activity score, macrophage infiltration, and LPS accumulation, indicating an improvement of inflammation. Furthermore, antibiotic-treated LDLR^-/-^ hamsters consistently showed less atherosclerotic lesions in the aortic roots and the whole aortas. Thus, we believe that antibiotics in our hamster model have the dual effect of lipid lowering and anti-inflammation to protect from diet-induced NASH and atherosclerosis. It is of note that in contrast to the previous findings reported in WT hamster model, we found that bacterial translocation from intestine to liver was effectively inhibited; however, the number of systemic immune cells were not substantially affected after antibiotic treatment in LDLR^-/-^ hamsters. These discrepancies were probably caused by the dietary composition, the genotypes and even the time duration for the different experimental purposes. Although antibiotics primarily target the gut microbiota, it is worth noting that metronidazole, ampicillin, and kanamycin are absorbable antibiotics [22]. In the present study, we did not measure the plasma concentrations of these antibiotics. Therefore, it cannot be excluded that the antibiotics exert their anti-NAFLD and antiatherosclerosis effects in a gut microbiota-independent manner.

In addition, accumulation of excess fat has deleterious consequences for health [23]. BAT activation and white adipose beige, facilitating thermogenesis to maintain host core body temperature, have become a trending topic on an antiobesity and associated metabolic disorders [24]. Li and colleagues [25] found that UCP1 expression in BAT was decreased, thermogenesis was impaired, and WAT browning was blocked in gut microbiota depletion mice treated with different antibiotic cocktails, leading to a lower in core body temperature. However, in the present study, we found that antibiotic treatment promoted thermogenesis by increasing UCP1 expression in BAT, facilitating the browning process of WAT, and then increasing rectal core body temperature, to protect against HFHC diet-induced obesity in antibiotic-treated LDLR^-/-^ hamsters. Thus, based on the different observations between mice and hamsters, in which intestinal microbiota has been depleted in the former, whereas reshaped in the latter, we speculated that antibiotics execute an indirect function on thermogenesis and antiobesity effect in a microbiota-dependent manner.

Nevertheless, we did not observe any detrimental effects of antibiotic treatment in our study. Long-term antibiotic treatment has been reported to cause many side effects, including memory deficits, pain, digestive disease, and antibiotic resistance [26,27], suggesting that screening new metabolites with specific bioactivities and safety under antibiotic administration, which will be applied to the treatment of CHL disease, should be paid attention. Using untargeted metabolomic analysis, we found that the abundance of the synthetic enzymes responsible for the productions of BA and GG were markedly increased, making BA and GG be the 2 top metabolites in LDLR^-/-^ hamsters with antibiotic treatment. The oral administration of BA and GG, respectively, could substantially ameliorate NASH and atherosclerosis in HFHC diet-fed LDLR^-/-^ hamsters with the former more potent. As one of the major SCFAs, BA supplementation not only reduced plasma TG, TC, and glucose levels with an increase in HDL-C level but also modulated immune response to suppress the inflammation, eventually improving NASH and atherosclerosis in HFHC diet-fed LDLR^-/-^ hamsters showing severe CHL and demonstrating that BA could be a potential therapeutic approach to treat CHL disease. Unlike our data collected from the LDLR^-/-^ hamster model, most of the previous work studied the role of BA in metabolic disease in ApoE^-/-^ mice, a mouse model prone to diet-induced hypercholesterolemia and atherogenesis. Consistently, BA or BA producers can suppress the inflammation to slow down atherosclerotic development [28–30], whereas their influence on lipid metabolism is still controversial. Two independent groups reported that BA decreased plasma TC levels and increased HDL-C levels without affecting plasma TG levels in HFD-fed ApoE^-/-^ mice [31,32]; however, Ma et al. [33] later showed that BA effectively lowered both TC and TG levels but did not increase HDL-C levels. Recently, using ApoE*3 Leiden.CETP mice, a mouse model generated to better mimic human, Liu and colleagues [34] found that BA had no effects on cholesterol metabolism and atherosclerosis, but the data of TG metabolism had not been reported in this human-like mouse model. Thus, it will be tempting to make a head-to-head comparison to understand the function of SCFAs in lipid metabolism and the metabolic disease in the different species in future.

Recently, perturbation in amino acid metabolism has been implicated in NASH [35–37]. Impaired glycine metabolism has been uncovered as a causative factor and therapeutic target in NASH and related cardiometabolic diseases [38–40]. Unlike BA that execute dual functions, GG, a dipeptide (Gly-Gly), relieves NASH (steatohepatitis, inflammation, and fibrosis) and atherosclerosis in LDLR^-/-^ hamsters to some extent without altering lipid profile in circulation, which is very similar to the recent findings reported in nonhuman primate NASH model treated with tripeptide DT-109 (Gly-Gly-Leu) through its anti-inflammatory property [41], suggesting the importance of testing glycine-based drugs in clinical trials as a potential treatment option for CHL-associated NAFLD and CVD.

Previous research has illustrated that BA mitigates HFD-induced steatohepatitis in C57BL/6J mice by enhancing gut microbiota and fortifying the gastrointestinal barrier [42]. Conversely, another study has suggested that BA safeguards against diet-induced NASH and liver fibrosis in LDLR^-/-^*Leiden mice by rectifying hyperinsulinemia, reducing plasma leptin levels, and alleviating adipose tissue inflammation, without influencing gut permeability or microbiota composition [43]. Later, Beisner and the colleagues [44] have contributed to the valuable insights that BA alleviates hepatic steatosis in Western-style diet-fed C57BL/6 mice, which is attributable to the induction of Paneth cell α-defensins and matrix metalloproteinase-7 via histone deacetylation and signal transducer and activator of transcription 3. Collectively, these findings propose that BA holds the potential to ameliorate metabolic diseases through both microbiota-dependent or -independent pathways. Recently, GG has garnered recognition for its protective functions in liver preservation [45]. It is noteworthy that glycine, the hydrolytic byproduct derived from GG, has been shown to exert a beneficial influence on NAFLD by orchestrating modifications in the intestinal flora [38,41], which underscores the multifaceted nature of GG’s effects, implicating its potential role in addressing liver-related concerns. As expected, in the present study, we indeed observed that supplementary BA and GG indeed substantially mitigated diet-induced atherosclerosis and NASH in LDLR^-/-^ hamsters, suggesting that these 2 compounds could be considered to be applied to the treatment of CHL-associated diseases. However, it will be tempting for us to further investigate the precise molecular mechanism by which BA and GG protect against atherosclerosis and NASH and whether the altered gut microbiome contributes to the protective effects of BA and GG and in the further study.

In summary, our study is the first to show that remodeling the gut microbiota with intermittent antibiotic treatment and subsequent transfer via cohousing, and supplementing with beneficial microbial metabolites effectively alleviate NASH and atherosclerosis in both HFHC and HC diet-fed LDLR^-/-^ hamster models, closely resembling CHL patient characteristics. These findings suggest that such interventions hold promise as potential therapeutic strategies for addressing CHL-associated metabolic disorders.

## Materials and Methods

Detailed methods are provided below and within the Supplementary Methods in the Supplementary Materials for all procedures carried out in this study. Biological replicates are incorporated for all datasets in this study. The datasets that support the findings of the study are available from the corresponding author upon reasonable request (xianxunde@bjmu.edu.cn).

### Animals

LDLR^-/-^ Syrian golden hamsters were generated in our laboratory using the CRISPR/Cas9 gene editing system as described previously [46]. Male LDLR^-/-^ hamsters were specifically chosen for our study due to the male gender being a substantial risk factor for CHL, aligning with human scenarios [47,48]. The 42 2-month-old male LDLR^-/-^ hamsters were randomly divided into 7 groups, each group containing 6 hamsters: PS (placebo, separately housed), AS (antibiotic, separately housed), PM (placebo, cohoused with antibiotic-treated hamsters), AM (antibiotic, cohoused with placebo-treated hamsters), CON (control water), BA (150 mM sodium BA in drinking water), and GG (1 mg/kg body weight/d GG by gavage once a day). Maintained on a 14-h light/10-h dark cycle at 24 °C, LDLR^-/-^ hamsters were subjected to either an HFHC diet (0.5% cholesterol and 20% lard [w/w]) or an HC diet (0.05% cholesterol [w/w]) based on a powdered chow diet processed by BiotechHD Co. LTD., Beijing, China, with water ad libitum. For HFHC diet-fed hamsters, a combination of antibiotics was vancomycin (50 mg/kg, HY-17362), metronidazole (100 mg/kg, HY-B0318), kanamycin (100 mg/kg, HY-16566A), and ampicillin (100 mg/kg, HY-B0522A). On the HC diet, antibiotic dosages were halved. Sodium BA (156-54-7) and GG (S20163) were purchased from Shanghai Yuanye Biotechnology Co., LTD. All antibiotics were purchased from MCE. Fresh solutions were prepared for each administration to promise its activity. LDLR^-/-^ hamsters received antibiotics via gavage at 9 AM every Monday and Friday for 8 weeks.

Hamsters were anesthetized under isoflurane inhalation (3%) followed by cervical dislocation. Liver, adipose tissue, aorta, heart, and blood samples were collected. Unless stated otherwise, hamsters were fasted for 12 h before euthanasia. LDLR^-/-^ hamsters that did not survive to the end of the experiment were excluded. All experiments followed the principle of experimental animal care (NIH publication no.85Y23, revised 1996) and were approved by the Animal Care and Use Committee of Peking University (LA2022147, Beijing, China).

### Statistical analysis

All data were presented as means ± SEM. The normality of the distribution of examined parameters was evaluated using the Shapiro–Wilk test. All statistical analysis was conducted using GraphPad Prism 8.0 software. For comparison between 2 groups, the unpaired *t* test was employed, while the comparison among multiple groups was performed using 1- or 2-way analysis of variance (ANOVA) followed by the Tukey’s multiple comparisons test. A *P* value less than 0.05 was deemed statistically substantial.

## Data Availability

All data are present in the paper and/or the Supplementary Materials.
