# Supplementary material for: Remodeling Intestinal Microbiota Alleviates Severe Combined Hyperlipidemia-Induced Nonalcoholic Steatohepatitis and Atherosclerosis in LDLR-/- Hamsters
Source: Research (Wash D C). 2024 Apr 29;7:0363. doi: 10.34133/research.0363 (PMC11062505; doi:10.34133/research.0363)
Supplement: Supplementary 1 — Supplementary Methods Figs. S1 to S7 Table S1 [file research.0363.f1.docx]

**Supplementary Methods**

**1. Microbiota analysis**

Fresh feces of hamsters from indicated groups were collected. The CTAB/SDS method was used to extract the total genome DNA in samples. DNA concentration and purity were monitored on 1% agarose gel. According to the concentration, DNA was diluted to 1ng/µL with sterile water. 16S ribosomal DNA V4 was amplified with specific primer and barcodes. All PCR mixture contained 15 µL of Phusion® High-Fidelity PCR Master Mix (New England Biolabs), 0.2 µM of each primer, and 10 ng target DNA, and cycling conditions consisted of a first denaturation step at 98 °C for 1 min, followed by 30 cycles at 98 °C (10 s), 50 °C (the 30 s) and 72 °C (30 s) and a final 5 min extension at 72 °C. Following the manufacturer’s recommendations, sequencing libraries were generated with NEBNext® Ultra™ IIDNA Library Prep Kit (Cat No. E7645). The library quality was evaluated on the Qubit@ 2.0 Fluorometer (Thermo Scientific) and Agilent Bioanalyzer 2100 system. Finally, the library was sequenced on an Illumina NovaSeq platform and 250 bp paired-end reads were generated. Paired-end reads were assigned to samples based on their unique barcode and truncated by cutting off the barcode and primer sequence. Paired-end reads were merged using FLASH (Version 1.2.11, http://ccb.jhu.edu/software/FLASH/), a very fast and accurate analysis tool designed to merge paired-end reads when at least some of the reads overlap with the reads generated from the opposite end of the same DNA fragment, and the splicing sequences were called raw tags. Quality filtering on the raw tags were performed using the fastp (Version 0.20.0) software to obtain high-quality clean tags. The clean tags were compared with the reference database (Silva database https://www.arb-silva.de/ for 16S/18S) using Vsearch (Version 2.15.0) to detect the chimera sequences, and then the chimera sequences were removed to obtain the effective tags. To analyze the diversity, richness, and uniformity of the communities in the sample, alpha diversity was calculated in QIIME2. Beta diversity was calculated based on weighted and unweighted unifrac distances in QIIME2 to evaluate the complexity of the community composition and compare the differences between groups. Cluster analysis was preceded by principal component analysis (PCA), which was applied to reduce the dimension of the original variables using the ade4 package and ggplot2package with R software (Version 4.0.3). To study the significance of the differences in community structure between groups, the adonis and anosim functions in the QIIME2 software were used to do analysis. To find out the significantly different species at each taxonomic level (Phylum, Class, Order, Family, Genus, Species), the R software (Version 3.5.3) was used to do MetaStat and T-test analysis. The LEfSe software (Version 1.0) was used to do LEfSe analysis (LDA score threshold: 4) to find out the biomarkers. Further, to study the functions of the communities in the samples and find out the different functions of the communities in the different groups, the PICRUSt2 software (Version 2.1.2-b) was used for functional annotation analysis.

**2 Untargeted metabolomic analysis**

**2.1 Metabolite extraction**

Feces or liver tissues (100 mg) were individually grounded with liquid nitrogen and the homogenate was resuspended with prechilled 80% methanol by a well vortex. The samples were incubated on ice for 5 min and then were centrifuged at 15,000 g, 4 °C for 20 min. Some of the supernatant was diluted to a final concentration containing 53% methanol by LC-MS grade water. The samples were subsequently transferred to a fresh Eppendorf tube and then were centrifuged at 15000 g, 4 °C for 20 min. Finally, the supernatant was injected into the UHPLC-MS/MS system analysis.

**2.2 UHPLC-MS/MS analysis**

UHPLC-MS/MS analyses were performed using a Vanquish UHPLC system (Thermo Fisher, Germany) coupled with an Orbitrap Q ExactiveTMHF-X mass spectrometer (Thermo Fisher, Germany) in Novogene Co., Ltd. (Beijing, China). Samples were injected onto a Hypesil Gold column (100×2.1 mm, 1.9 μm) using a 12-min linear gradient at a flow rate of 0.2mL/min. The eluents for the positive polarity mode were eluent A (0.1% FA in Water) and eluent B (Methanol). The eluents for the negative polarity mode were eluent A (5 mM ammonium acetate, pH 9.0) and eluent B (Methanol). The solvent gradient was set as follows: 2% B, 1.5 min; 2-85% B, 3 min; 85-100% B, 10 min; 100-2% B, 10.1 min;2% B, 12 min. Q ExactiveTM HF-X mass spectrometer was operated in positive/negative polarity mode with a spray voltage of 3.5 kV, a capillary temperature of 320 °C, sheath gas flow rate of 35 psi, and aux gas flow rate of 10 L/min, S-lens RF level of 60, Aux gas heater temperature of 350 °C.

**2.3 Data analysis**

The raw data files generated by UHPLC-MS/MS were processed using Compound Discoverer 3.1 (CD3.1, Thermo Fisher) to perform peak alignment, peak picking, and quantitation for each metabolite. The main parameters were set as follows: retention time tolerance, 0.2 min; actual mass tolerance, 5ppm; signal intensity tolerance, 30%; signal/noise ratio, 3; and minimum intensity, et al. After that, peak intensities were normalized to the total spectral intensity. The normalized data were used to predict the molecular formula based on additive ions, molecular ion peaks, and fragment ions. Then peaks were matched with the mzCloud (<https://www.mzcloud.org/>), mzVault, and MassList databases to obtain accurate qualitative and relative quantitative results. Principal Component Analysis (PCA) was employed to reduce the dimensionality of the original variables using the ade4 package and ggplot2 package in R software (Version 4.0.3). The resulting PCA plot illustrates sample distribution based on the scores of the first (PC1) and second (PC2) principal components. Each point on the plot represents a sample, with distinct colors representing different subgroups. Tighter clustering within a group indicates greater internal consistency, while increased separation between groups signifies more pronounced differences, as depicted in the accompanying figure.

Utilizing a hypergeometric test, we derived p-values for pathway enrichment, considering a threshold of p-value ≤ 0.05. KEGG pathways meeting this criterion were identified as significantly enriched in differential metabolites. The x/y coordinates in the KEGG enrichment bubble plot represent the ratio of differential metabolites to the total metabolites identified in the corresponding pathway, with a higher value indicating a more pronounced enrichment of differential metabolites. The color of the bubbles reflects the p-value obtained from the hypergeometric test; smaller values signify greater reliability and statistical significance. The size of the bubbles correlates with the number of differential metabolites in the respective pathway, with larger sizes denoting a higher abundance of differential metabolites.

**3. Western blot (WB)**

Plasma samples were mixed with 5× SDS-PAGE loading buffer (P0015L, Beyotime, China) and denatured at 95 °C for 10 min. Subsequently, the samples were analyzed via SDS-PAGE gels and transferred onto Nitrocellulose membranes. Following the transfer, the membranes were blocked with 5% non-fat milk in TBST buffer (25 mM Tris, 137 mM NaCl, 2.7 mM KCl, 0.075% Tween-20) at room temperature for 1 hour, which were then incubated with the specific primary antibody in TBST buffer containing 5% BSA at 4 °C overnight. After incubation, the membranes were thoroughly rinsed and exposed to a horseradish peroxidase-conjugated secondary antibody (ZSGB-BIO, China) in TBST buffer supplemented with 5% BSA at room temperature for 1 hour. Visualization of the target proteins was achieved using Pierce ECL Plus Western blotting substrate (32209, Thermo Fisher Scientific, USA).

**4. Test of blood biochemical parameters**

Plasma samples were collected from the retro-orbital plexus of the hamsters after 12-hour fasting. Non-esterified fatty acid (NEFA) was measured with a commercial enzymatic kit (Wako, Japan). The degree of liver injury was estimated based on the plasma alanine aminotransferase (ALT) and aspartate transaminase (AST) levels (Nanjing Jiancheng Bioengineering Institute, Nanjing, China).

**5. Analysis of plasma lipids, lipoproteins, and tissue lipid content**

The plasma total cholesterol (TC) and triglyceride (TG) levels were determined by enzymatic methods (Biosino Bio Technology & Science, Beijing, China). High-density lipoprotein cholesterol (HDL-C) was measured with the same TC kit after precipitating ApoB-containing lipoprotein by 20% polyethylene glycol.

To analyze the lipid distribution, fast protein liquid chromatography (FPLC) of plasma lipoproteins was performed. Pool plasma of each group (100μl/group after being filtered by 0.22-mm filter) was subjected to Tricorn high-performance Superose S-6 10/300GL column (Amersham Biosciences, Little Chalfont, Buckinghamshire, UK), eluting with PBS at a constant flow rate at 0.5 mL/min.

25 mg tissue samples (liver or feces) were homogenized in 1mL cold phosphate buffer solution (PBS), and then 4 mL of chloroform/methanol (v:v = 2:1) was added. The mixture was vortexed for 2 min and then allowed to stand at room temperature for 30 min. After centrifugation at 3000 rpm for 30 min, the chloroform layer was transferred to a new glass tube using a glass syringe and dried under a nitrogen stream. Lipids were dissolved with 500 μL of 5% triton X-100, and the contents of cholesterol and triglyceride were measured according to the method described above.

**6. Pathological analysis**

The animals were sacrificed and perfused with cold PBS at the endpoint. The heart, liver, intestine, white adipose tissue (WAT), brown adipose tissue (BAT), and aorta were harvested and then fixed in 4% paraformaldehyde overnight, followed by 20% sucrose solution for dehydration. The heart, liver, and intestine were embedded in O.C.T solution. Liver, WAT and BAT were embedded in paraffin.

**6.1 Atherosclerotic lesion characterization**

The entire aortas (from the ascending aorta to the iliac artery bifurcation) were collected at the endpoints of the experiments, thoroughly cleaned under a dissecting microscope, and then fixed with 4% paraformaldehyde. *En face* was stained with oil red O (O1319, Sigma, Germany). Hearts were embedded in O.C.T (4583, Sakura, USA) and sectioned (7-μm thickness) from the first 3 aortic valves appeared until the 3 leaflets disappeared. The lesion areas in the aortic root were quantified by hematoxylin (HHS128-4L; Sigma, Germany) and eosin (HT110232-1L; Sigma, Germany) (H&E) staining. Neutral lipids deposition was determined by oil red O staining. The aortic lesion size and lipid content of each animal were obtained by an average of three sections from the same hamster. The lesion areas on a total of 6 sections through the aortic root were measured. The mean areas of the 6 sections/hamster were used for comparing lesion size between groups.

**6.2** **H&E and Picro sirius red staining:**

The paraffin-embedded tissue samples were sectioned, dewaxed, and rehydrated. Harry's hematoxylin was used for H&E staining for 5 min, followed by aqueous eosin for 3 min. The samples were then dehydrated, permeabilized, and sealed with gum. For Sirius red (R21890, Saint-Bio, China) staining, sections were stained with 0.01% Fast Green FCF in saturated picric acid for 15 min, followed by 0.04% Fast Green and 0.1% Sirius red in saturated picric acid for 15 min in accordance with the manufacturer’s procedure.

**6.3** **Immunohistochemical (IHC) staining**

The sections of paraffin-embedded liver, BAT, and WAT tissue from LDLR^-/-^ hamsters underwent dewaxing, rehydration, antigen unmasking, and endogenous peroxidase blockade, followed by an incubation with goat serum (ZLI-9022, ZSGB-BIO, China) for 1 hour at 37 °C. Afterwards, LPS antibody (MAB526Ge21, Cloud-Clone Corp, China) to the liver sections, UCP1 antibody (A5857, ABclonal, China) to BAT sections and CD68 antibody (BM3639, BOSTER, China) to the WAT sections were applied for overnight incubation. As subsequent incubation with secondary antibody (ZSGB-BIO, China) for 1 hour at 37 °C,1x diaminobenzidine (DAB, ZLI-9018, ZSGB-BIO, China) was used to visualize the signals for 90-120 seconds in the sections that were counterstained with hematoxylin, dehydrated, and finally mounted with coverslips.

**6.4** **Immunofluorescence (IF) staining**

Frozen liver sections were fixed with 4% paraformaldehyde for 30 min. For permeability, the sections were incubated with PBS containing 0.1% (v/v) Triton X-100 for 15 min. After blocking in PBS with 3% (w/v) BSA, CD68 antibody was incubated in the blocking buffer overnight at 4 °C. Afterwards, the sections were washed with PBS containing 0.1% (v/v) Tween 20 and then incubated with Alexa Fluor-conjugated secondary antibodies (ab150115, Abcam, USA) for 1 hour at 37 °C in darkness at 1:1000 dilution. BODIPY was incubated at 4 °C for 30 min. DAPI (C1005, Beyotime, China) or BODIPY dye (D3922, Invitrogen, USA) were also used to display nuclei and lipid droplets respectively.

**6.5 Image analysis:**

H&E, Picro sirius red, or oil red O staining were analyzed by Leica DM3000 upright light microscope. The slides were scanned using Grundium Ocus® microscope slide scanners (Tampere, Finland) for presentative pictures. The calculation of intensity and positive area was processed by Image-Pro Plus 6.0 software for quantitation. Super-resolution fluorescent imaging was conducted by All-in-One Fluorescence Microscope BZ-X810 (KEYENCE, USA) with BZ-H4A/Advanced Analysis Software. Image analysis and quantification were performed using Fiji software (9.0).

**7. RNA isolation and quantitative real-time PCR**

Total RNA was extracted from different tissues by Trizol reagent (ER501, Transgen Biotech, China) and the first-strand cDNA was reversely transcribed using a reverse transcription kit (AT411, Transgen Biotech, China). Quantitative real-time PCR was performed using primers listed in Extended Data Table S1. The amplification reactions were performed using the Mx3000 Multiplex Quantitative PCR System (40 cycles: denaturation at 94 °C for 15 s, annealing at 60 °C for the 20 s, and extension at 72 °C for 45 s). All data of gene expression were normalized to the PS group.

**8. Metabolic assays**

**8.1 Oral fat load**

Hamsters were gavaged with olive oil (10 mg/kg body weight) after 12-hour fasting. Plasma samples were collected at the indicated time points (0 h, 0.5 h, 1 h, 2 h, 4 h and 8 h) after gavage for plasma TG measurement.

**8.2 LPL activity assay**

Plasma was collected 30 min after intraperitoneal injection of heparin (2000 U/kg body weight). LPL activity was measured using a commercial LPL activity kit (ab204721, Abcam, USA) and presented by free fatty acid release (pmol/mL/min).

**8.3 Glucose tolerance tests and insulin tolerance tests** **assays**

For the glucose tolerance test, the hamsters were fasted for 16 h overnight. The blood glucose was measured at different time points (0, 15, 30, 60, 90 and 120 min) before and after glucose (2.5 g/kg) challenge. The insulin tolerance test was carried out after fasting for 6 h. Blood glucose was measured before and after (0, 15, 30, 60 and 90 min) intraperitoneal injections of insulin (1.5 UI/kg) by glucose kits (Biosino Bio Technology & Science, Beijing, China).

**Supplementary Figures and Figure Legends**

**
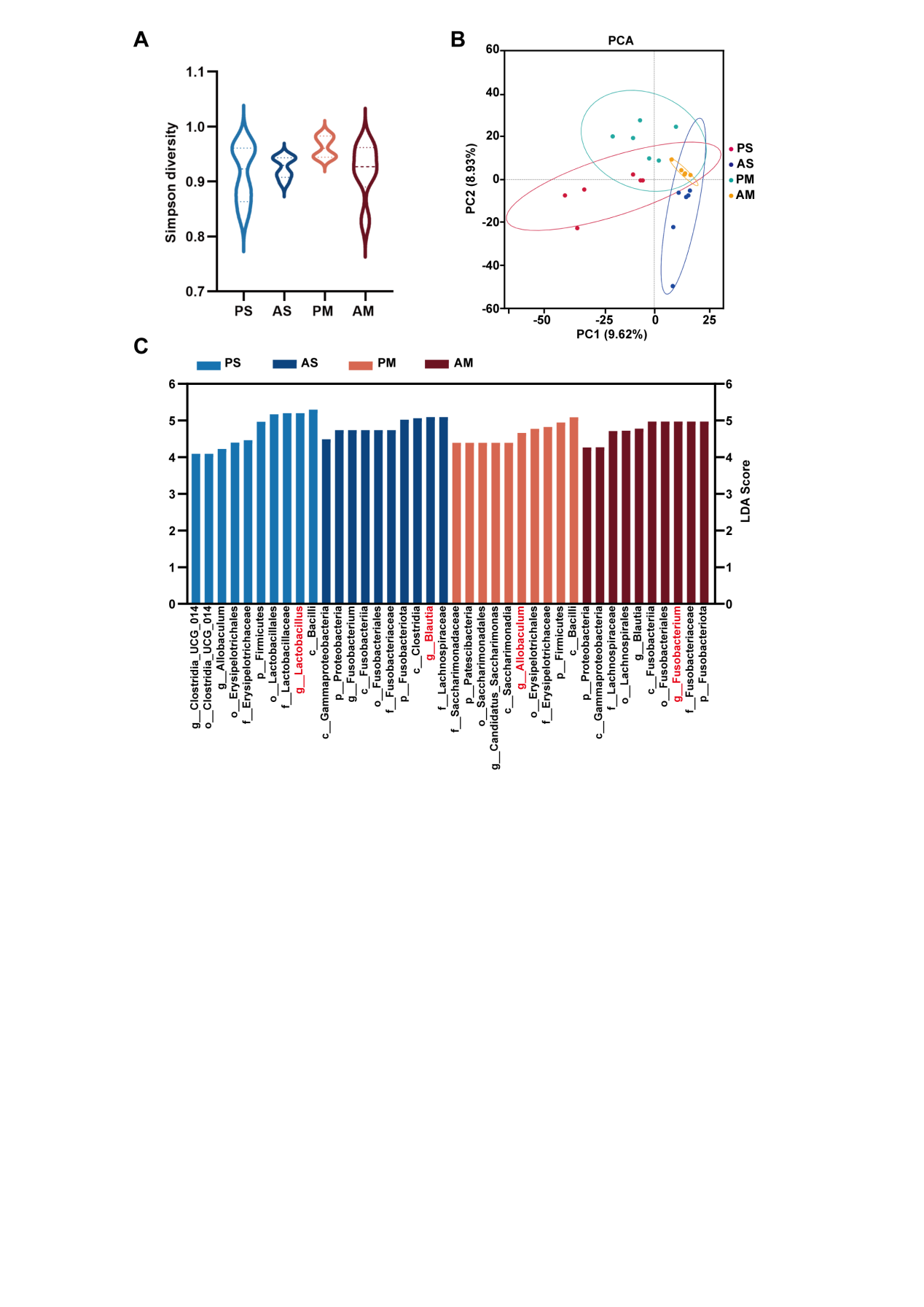
**

**Supplementary Figure 1. Antibiotics treatment reconstructed gut**

**microbiota communities.**

The LDLR^-/-^ hamsters fed HFHC diet were given a placebo or antibiotics for 8

weeks by a separate or cohousing approach (n = 6/group). **A**-**C**. Simpson

diversity (**A**), Principal component analysis (**B**), and Linear discriminant

analysis (LDA) scores (**C**) of gut microbiota among indicated four groups of

LDLR^-/-^ hamsters. PS, LDLR^-/-^ hamsters given placebo were separately

housed; AS, LDLR^-/-^ hamsters with antibiotic treatment were separately

housed; PM, LDLR^-/-^ hamsters given placebo were cohoused with LDLR^-/-^

hamsters given antibiotics; AM, LDLR^-/-^ hamsters given antibiotics were

cohoused with LDLR^-/-^ hamsters given placebo.


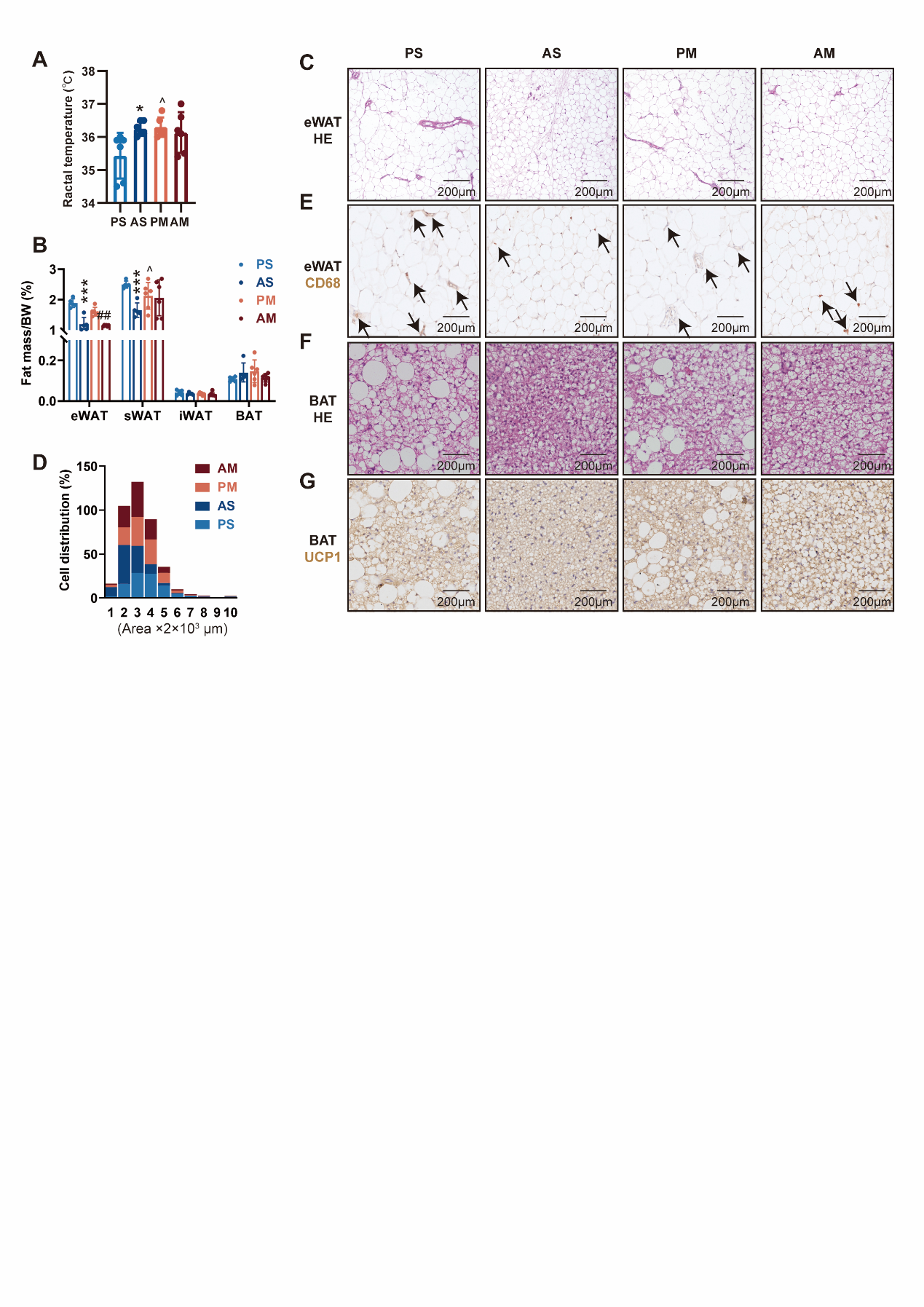


**Supplementary Figure 2. Antibiotics treatment prevented HFHC diet-**

**induced adipocyte hypertrophy and macrophage infiltration in LDLR**^-/-^

**hamsters.**

The LDLR^-/-^ hamsters fed HFHC diet were given a placebo or antibiotics for 8

weeks by a separate or cohousing approach (n = 6/group). **A.** Rectal core body

temperature of LDLR^-/-^ hamsters at 24 °C incubator. **B**. The ratio of fat mass to

body weight in epididymal adipose tissue (eWAT), subcutaneous adipose

tissue (sWAT), inguinal adipose tissue (iWAT), and brown adipose tissue (BAT). **C**. H&E staining of eWAT from different groups. **D**. Measurements of adipocyte area of eWAT from PS, AS, PM, AM group. The quantification is performed using Image J 8.0 software. Each plot represents a distribution of an individual adipocyte population according to size (area). Each distribution is obtained from six LDLR^-/-^ hamsters in each group and at least 100 adipocytes in each hamster. **E**. Immunohistochemical staining of CD68 in eWAT from different groups. Positive immunoreactivity was observed as a brown precipitate. **F**. H&E staining of BAT from different groups. **G**. Immunohistochemical staining of UCP1 in BAT from different groups. Positive immunoreactivity was observed as a brown precipitate. Data are expressed as mean ± SEM, analyzed by two-way ANOVA using Prism 8.0. **P* < 0.05, ****P* < 0.001 AS *vs* PS; ^^^*P* < 0.05 PM *vs* PS. PS, LDLR^-/-^ hamsters given placebo were separately housed; AS, LDLR^-/-^ hamsters with antibiotic treatment were separately housed; PM, LDLR^-/-^ hamsters given placebo were cohoused with LDLR^-/-^ hamsters given antibiotics; AM, LDLR^-/-^ hamsters given antibiotics were cohoused with LDLR^-/-^ hamsters given placebo.**
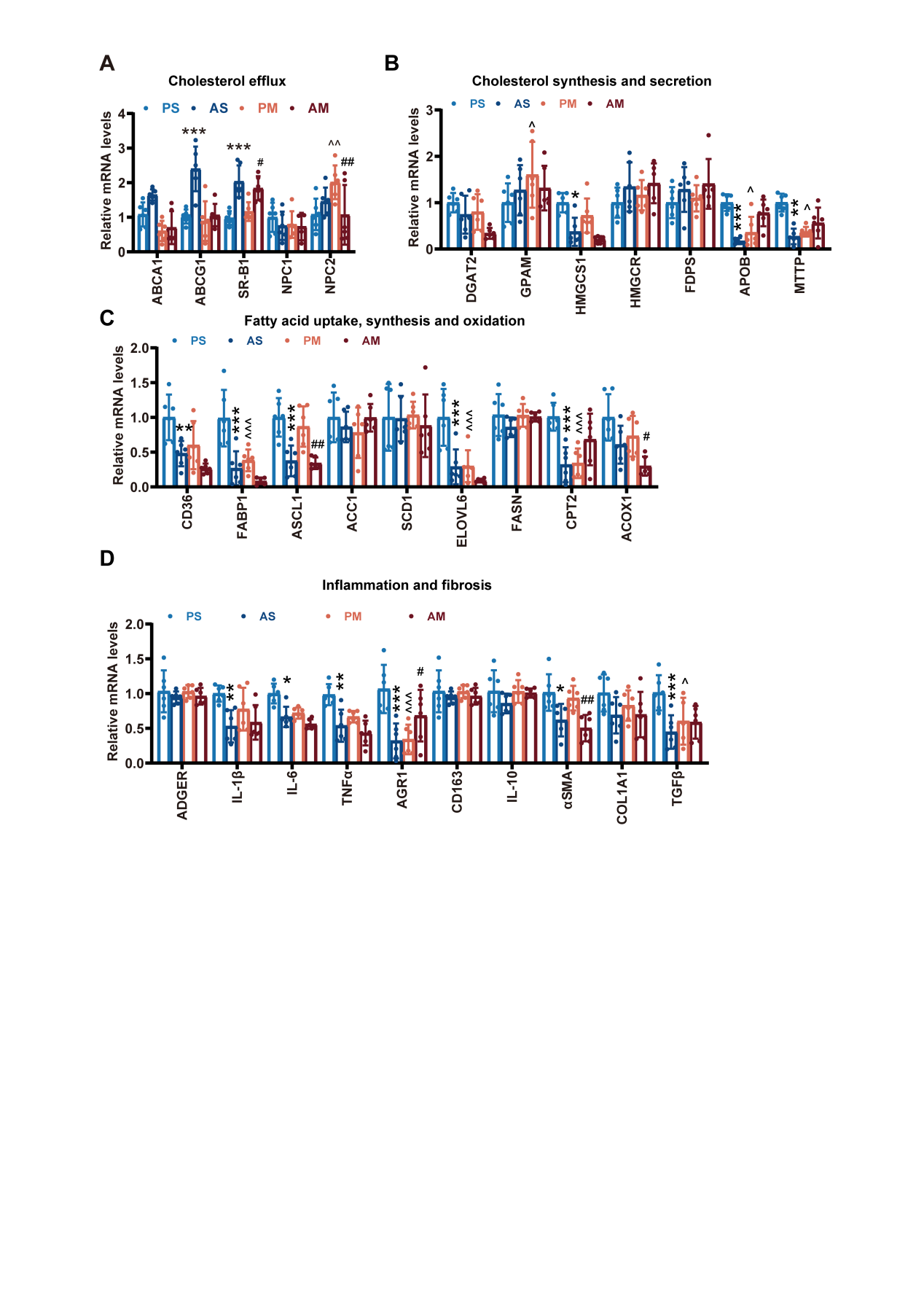
Supplementary Figure 3. Antibiotics treatment ameliorated HFHC diet-**

**induced NASH in LDLR^-/-^ hamsters.**

The LDLR^-/-^ hamsters fed the HFHC diet were given a placebo or antibiotics for 12 weeks by a separate or cohousing approach (n = 6/group). RNAs were extracted from livers and quantified by qPCR. **A**. mRNA expression of genes associated with cholesterol efflux. **B**. mRNA expression of genes involved in cholesterol synthesis and secretion. **C**. mRNA expression of genes related to fatty acid uptake, synthesis, and oxidation. **D**. mRNA expression levels of inflammation and fibrosis genes. Data are expressed as mean ± SEM, analyzed by two-way ANOVA using Prism 8.0. **P* < 0.05, ***P* < 0.01 and ****P* < 0.001 AS *vs* PS; ^^^*P* < 0.05, ^^^^*P* < 0.01, ^^^^^*P* < 0.001 PM *vs* PS; ^#^*P* < 0.05, ^##^*P* < 0.01, ^###^*P* < 0.001 AM *vs* PM. PS, LDLR^-/-^ hamsters given placebo were separately housed; AS, LDLR^-/-^ hamsters with antibiotic treatment were separately housed; PM, LDLR^-/-^ hamsters given placebo were cohoused with LDLR^-/-^ hamsters given antibiotics; AM, LDLR^-/-^ hamsters given antibiotics were cohoused with LDLR^-/-^ hamsters given placebo.


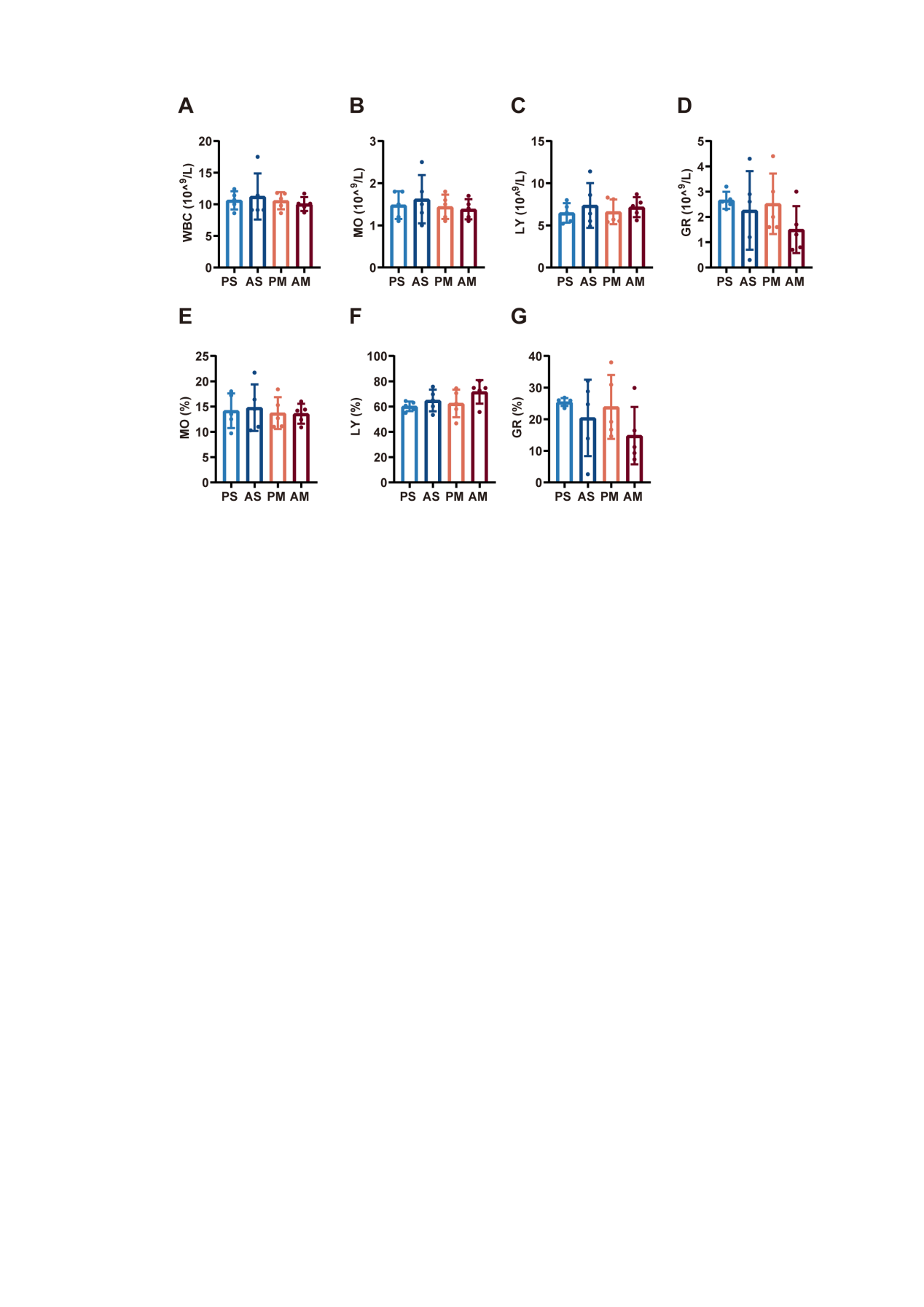
**Supplementary Figure 4. Impact of gut microbiota reshaping on**

**circulating immune cells**

LDLR^-/-^ hamsters were maintained on an HFHC diet for 4 weeks, and blood

samples were collected from the orbital vein to assess the influence of gut

microbiota reshaping on circulating immune cells (n = 5/group). **A**. White

blood cell numbers. **B**. Monocyte numbers. **C**. Lymphocyte numbers. **D**.

Granulocyte numbers. **E**. Percentage of monocytes. **F**. Percentage of

lymphocytes. **G**. Percentage of granulocytes. Data are analyzed by two-way

ANOVA using Prism 8.0. PS, LDLR^-/-^ hamsters given placebo were separately

housed; AS, LDLR^-/-^ hamsters with antibiotic treatment were separately

housed; PM, LDLR^-/-^ hamsters given placebo were cohoused with LDLR^-/-^

hamsters given antibiotics; AM, LDLR^-/-^ hamsters given antibiotics were

cohoused with LDLR^-/-^ hamsters given placebo.


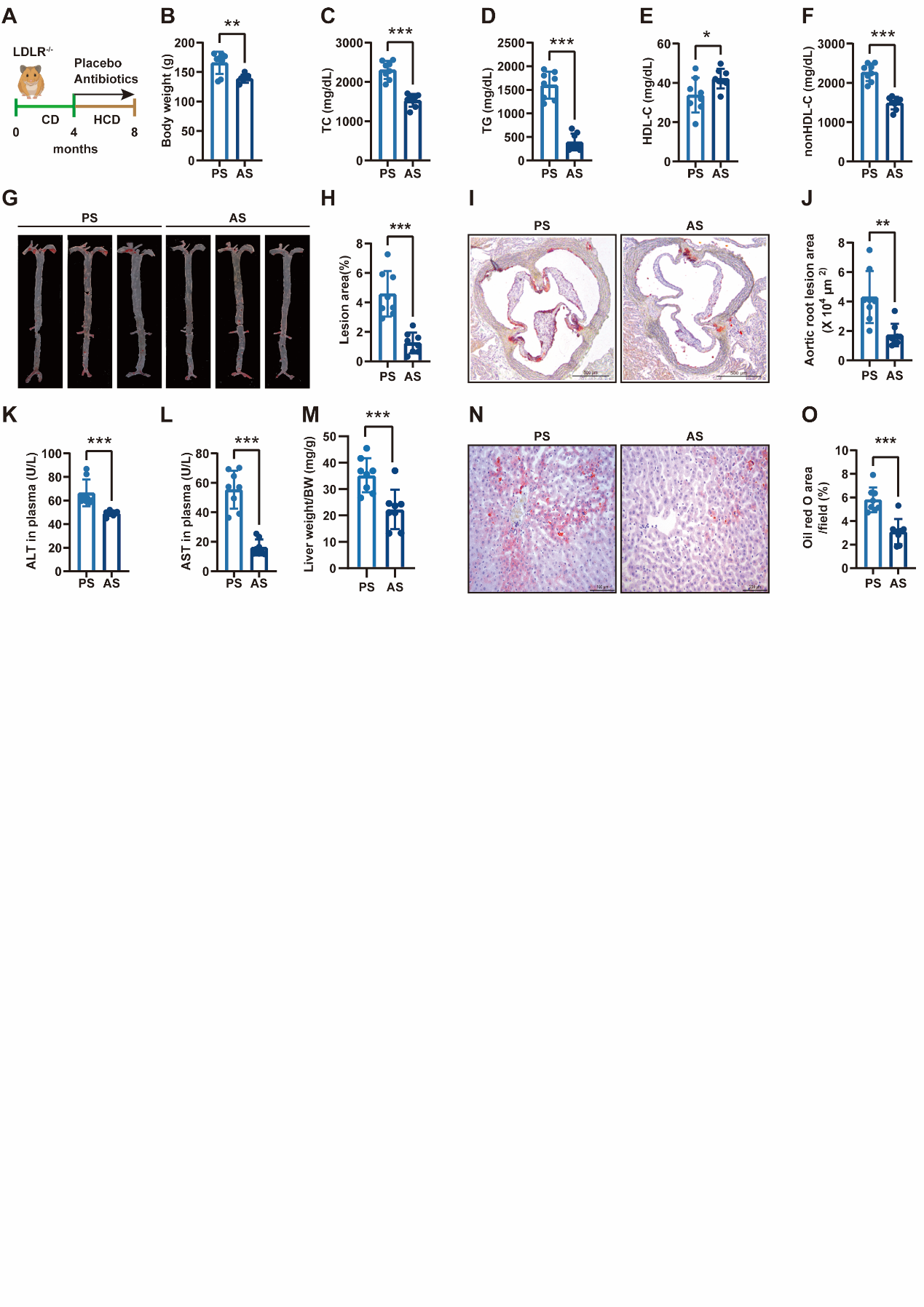


**Supplementary Figure 5. Antibiotic treatment attenuated HC diet-induced atherosclerosis and hepatic steatosis**

**A**. Experimental scheme depicting the entire study. Four-month-old male LDLR^-/-^ hamsters were subjected to a high-cholesterol (HC) diet containing 0.05% cholesterol for 4 months with either placebo or antibiotics, comprising vancomycin (25 mg/kg), metronidazole (50 mg/kg), kanamycin (50 mg/kg), and ampicillin (50 mg/kg). **B**-**F**. Body weight (**B**), the levels of plasma total cholesterol (TC) (**C**) triglyceride (TG) (**D**), high-density lipoprotein cholesterol (HDL-C) (**E**), and nonHDL-C (**F**). **G**-**H**. Representative *en face* images of atherosclerotic lesions in the entire aorta (**G**), and quantification of lesions (**H**). **I**-**J**. Representative images of the aortic root (**I**) and quantification of atherosclerotic lesions in the aortic root (**J**). **K**-**L**. Plasma alanine aminotransferase (ALT) (**K**) and aspartate aminotransferase (AST) (**L**) contents. **M**. The ratio of liver weight to body weight. **N**-**O**. Oil red O staining illustrating lipid droplets in the liver of LDLR^-/-^ hamsters (**N**), and quantification of liver lipid content (**O**). Data are analyzed by two-tailed Student’s t-test using Prism 8.0. **P* < 0.05, ***P* < 0.01 and ****P* < 0.001 AS *vs* PS. PS, LDLR^-/-^ hamsters given placebo were separately housed; AS, LDLR^-/-^ hamsters with antibiotic treatment were separately housed.

**
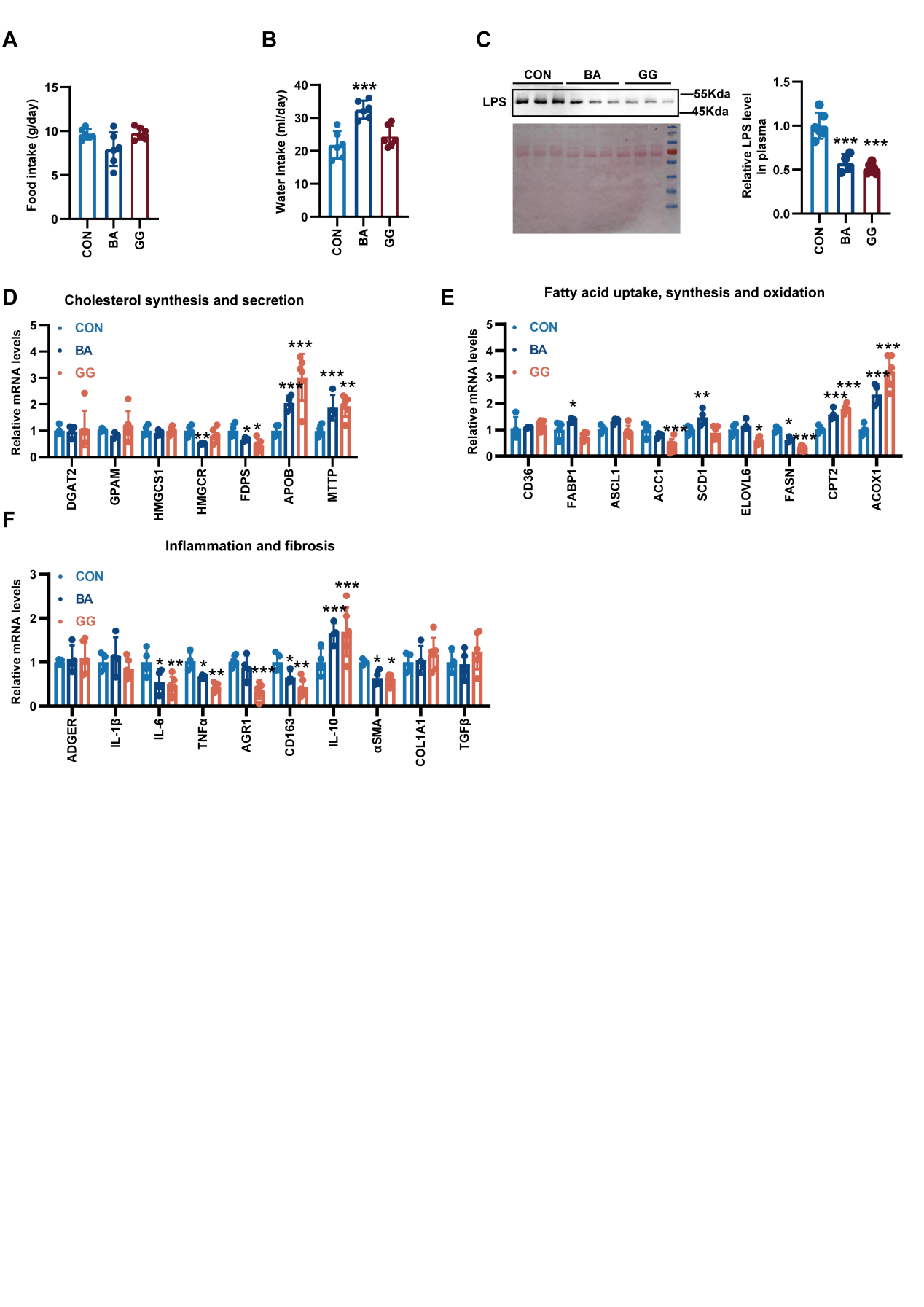
**

**Supplementary Figure 6. Effects of** **butyrate and glycylglycine treatment**

**on HFHC diet-induced NASH in LDLR^-/-^ hamsters.**

The LDLR^-/-^ hamsters fed the HFHC diet were given control water (CON),

sodium butyrate (BA), or glycylglycine (GG) for 8 weeks (n = 6/group). **A**. Food

intake. **B**. Water intake. **C**. Plasma Lipopolysaccharides (LPS) content. RNA

was extracted from livers and quantified by qPCR. **D**. mRNA expression of

genes involved in cholesterol synthesis and secretion in the liver. **E**. mRNA

expression of genes related to fatty acid uptake, synthesis, and oxidation in the

liver. **F**. mRNA expression levels of inflammation and fibrosis genes in the liver.

Data are expressed as mean ± SEM, analyzed by one-way ANOVA using

Prism 8.0. **P* < 0.05, ***P* < 0.01 and ****P* < 0.001 BA or GG *vs* CON. CON,

LDLR^-/-^ hamsters were given control water; BA, LDLR^-/-^ hamsters were given

sodium butyrate; GG, LDLR^-/-^ hamsters were given glycylglycine.


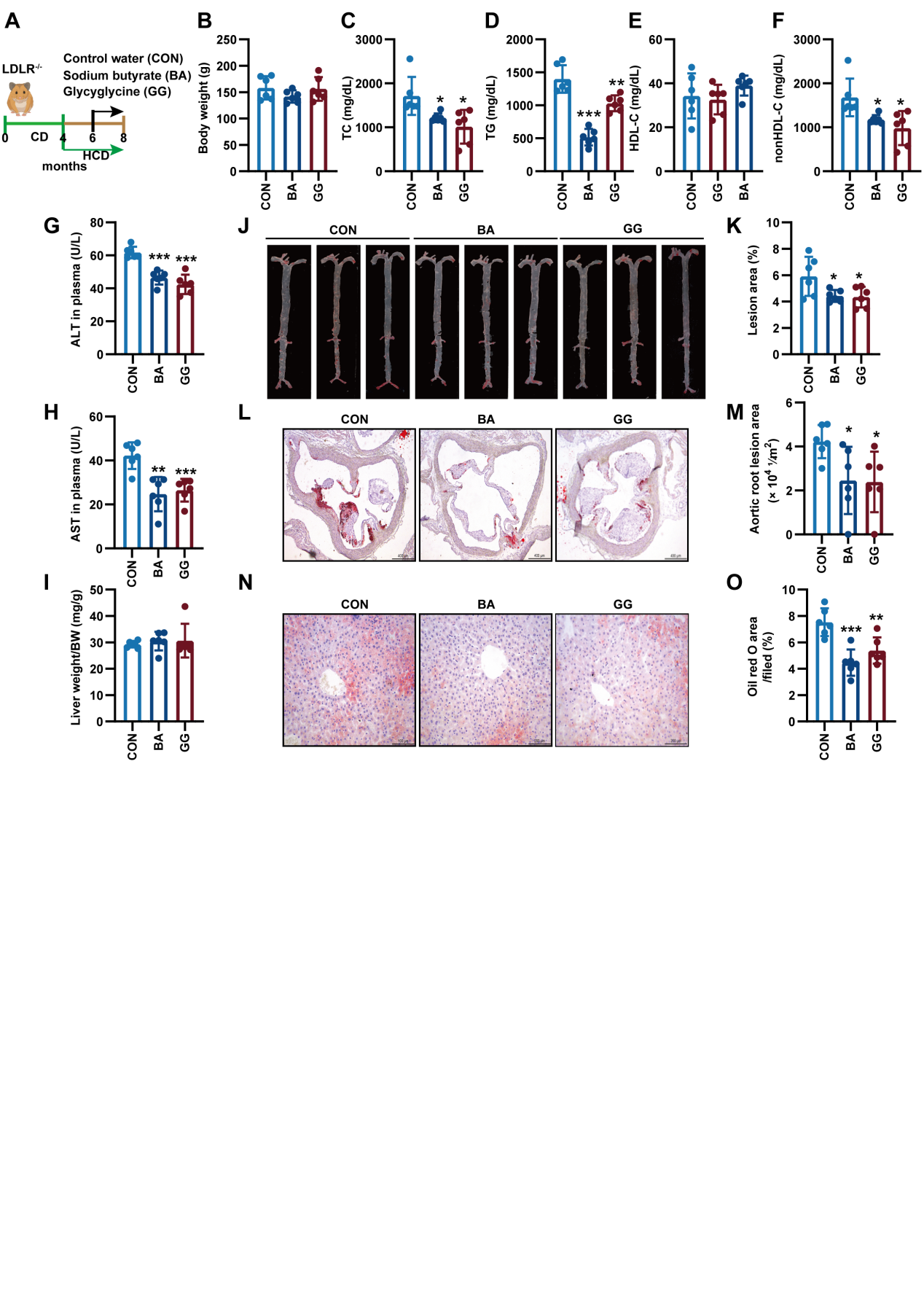


**Supplementary Figure 7. Alleviation of HC diet-induced hepatic steatosis**

**and atherosclerosis by butyrate and glycylglycine treatment.**

1. Experimental scheme outlining the comprehensive study. Four-month-old

male LDLR^-/-^ hamsters were fed the high-cholesterol (HC) diet containing 0.05% cholesterol for 4 months with control water (CON), sodium butyrate (BA), or glycylglycine (GG) for 8 weeks (n = 6/group). **B**-**F**. Body weight (**B**), the levels of plasma total cholesterol (TC) (**C**) triglyceride (TG) (**D**), high density lipoprotein cholesterol (HDL-C) (**E**), and nonHDL-C (**F**). **G**-**H**. Plasma

alanine aminotransferase (ALT) (**G**) and aspartate aminotransferase (AST) (**H**)

content. **I**. The ratio of liver weight to body weight. **J**-**K**. Representative images

of atherosclerotic lesions in the entire aorta (**J**) and quantification of lesions

(**K**). **L**-**M**. Representative images of the aortic root (**L**) and quantification of

atherosclerotic lesions in the aortic root (**M**). **N**-**O**. Oil red O staining depicting

lipid droplets in the liver of LDLR^-/-^ hamsters (**N**), and quantification of liver lipid

content (**O**)**.** Data are analyzed by one-way ANOVA using Prism 8.0. **P* < 0.05,

***P* < 0.01 and ****P* < 0.001 BA or GG *vs* CON. CON, LDLR^-/-^ hamsters were

given control water; BA, LDLR^-/-^ hamsters were given sodium butyrate; GG,

LDLR^-/-^ hamsters were given glycylglycine.

**Table S1. Hamster primer sequences for real-time PCR.**

| Gene Symbol | Forward Primer | Reverse Primer |
| --- | --- | --- |
| Zo-1 | ATGGGTAACTCCGTCCTCTGA | GTTAACTGAAGCACCACGGC |
| Ocln | TGATCGCTTGCCATCCACTT | TGATCGCTTGCCATCCACTT |
| Cdh1 | GCGTATCGGTGAGTTCTC | TAGACCTCGGCACTGAAG |
| Cldn1 | TTCTCGCTTCCAGATTCC | GGTCGTCGAGCAAGTTAT |
| Cldn2 | CTTACCAGGACTCACTCAG | ACAAGCAGGCTCAAGAAG |
| Cldn5 | ACACAAGAGACAGCACAG | TAACCTCATCCAAGATCCTAC |
| ABCA1 | GACACCTTCTACGACAACT | CATCTCTTGGCTGCTCTC |
| ABCG1 | GGTCAGTCCTCATCAATGG | TGCCTTCATCCTTCTCCT |
| SR-B1 | TCGGCTTCTGTCATCTCT | CGCACTATTGGCTTCTCA |
| NPC1 | AGTCTCTGCTGTGATGTTC | GTGCCTGTCCATTGTCTT |
| NPC2 | GAGCCTTCTTATCCACGAT | TAGAACCGCAGTCCTTGA |
| CD36 | TCATCTCTGTGGCCTCCGTC | AAGGATCATAACCACGCCGA |
| FABP1 | AGAAGCCCCTTATAAAATAGCCAC | GAGGTCATCAGGCAGACCAA |
| FABP2 | AGATCATGGCATTTGACGGGA | ATGAGTTGCAACCTTCCTTTTCA |
| FATP4 | GGGTCACAATGCTGCTTGGAG | GGAGAATCCCACCTGAGTCC |
| NPC1L1 | CATGTGTGGGGTGTATGGCT | CTGGTGTTGTGCGGTTTGAG |
| ADGER1 | CCTGCTATGTCGTGCTGTTCGT | GGCTGTCTGGTTGTCCGTCTTG |
| IL-1β | AGTCATTGTGGCTGTGGAGA | TGTTGTTCATCTCGGAGCCT |
| IL-6 | AGCCCACCAGGAACGAAAGACA | ACCAGCAGCAGTCCCAAGAAGA |
| TNFα | GCCACAATCCTCTTCTGCCT | GGAGCCGATGATAGGGTTGG |
| ARG1 | AGCTCCAAACCAAAGCCCATCG | TCCACCAGACCAGCATTCCTCA |
| CD163 | GAGCGGATCTGAGCCTGAGACT | GGCAGTGACAGCAGTTGGACAT |
| IL-10 | ACCTGGTAGAAGTGATGCCC | AGTTGCCTCCTGAGGGTCTT |
| αSMA | TGAGCGTGAGATTGTCCGAG | AAGCGTTCATTCCCGATGGT |
| COL1A1 | TGAAGGCAGCCGCAAGAACC | AGTCATGCTCTCGCCGAACCA |
| TGFβ | GACATGAGCACCATCCGTGACA | TCCGTGGAGCTGAAGCAGTAGT |
| DGAT2 | ATGAAGACCCTCATCGCTGC | CATTCTTGTTCTCGCTGCGG |
| GPAM | AAATGCAAACCGAAGGTGGC | GAGGCGCCATTATTTGCAGG |
| HMGCS1 | TGGAGGAACTGTCGGTGAGA | GTTGCAGAGCTAGTCACCGT |
| HMGCR | CACCCCTGGGAAGTTATTGTGG | CTGTTGTGCTGTTCTGAGGG |
| FDPS | CTCCTCTCTCAGAATGAATGGG | ATTGTACTTGCCTCCTACGGC |
| APOB | CAGTATTCTGCCAGCGCAAC | ACACTGCGGTAGCTTCAGTC |
| MTTP | CCTGGGAAGTGGATGCCTTA | CCTGGGAAGTGGATGCCTTA |
| ACSL1 | CAAACAGGTTGCAGAACGGG | CGCCAAGCGTATCGTAGAGT |
| ACC1 | GGCTATGTTGAGACGCTGGT | TGTGACTGGGCTTTGTGATCC |
| SCD1 | GGAGAAGCAGAAGACCGTTCC | CCCCTCCTCATCCTGGTAGC |
| ELOVL6 | TCTGATGAACAAGCGAGCGA | GACAGGTAAGAGGGAAGGGGT |
| FASN | CTCACACCCCCAAGATGGAC | ACTCCTTTGCCACCCGTATG |
| CPT2 | GAGCGATGCGTTAAAGGCTG | CATCATGTGCTGAAGCTCGC |
| ACOX1 | CGTCCGTCCCAAGAACTCC | GGGTCGTATGTGGCTGTAGT |
